# Acquisition of regulator on virulence plasmid of hypervirulent *Klebsiella* allows bacterial lifestyle switch in response to iron

**DOI:** 10.1128/mbio.01297-23

**Published:** 2023-08-02

**Authors:** Wilson H. W. Chu, Yi Han Tan, Si Yin Tan, Yahua Chen, Melvin Yong, David C. Lye, Shirin Kalimuddin, Sophia Archuleta, Yunn-Hwen Gan

**Affiliations:** 1 Infectious Diseases Translational Research Programme, Yong Loo Lin School of Medicine, National University of Singapore, Singapore, Singapore; 2 Department of Biochemistry, Yong Loo Lin School of Medicine, National University of Singapore, Singapore, Singapore; 3 National Centre for Infectious Diseases, Singapore, Singapore; 4 Tan Tock Seng Hospital, Singapore, Singapore; 5 Yong Loo Lin School of Medicine, National University of Singapore, Singapore, Singapore; 6 Lee Kong Chian School of Medicine, Nanyang Technological University, Singapore, Singapore; 7 Department of Infectious Diseases, Singapore General Hospital, Singapore, Singapore; 8 Program in Emerging Infectious Disease, Duke-NUS Medical School, Singapore, Singapore; 9 Division of Infectious Diseases, Department of Medicine, National University Hospital, National University Health System, Singapore, Singapore; 10 Department of Medicine, Yong Loo Lin School of Medicine, National University of Singapore, Singapore, Singapore; UCLA School of Medicine, Los Angeles, California, USA

**Keywords:** type 3 fimbriae, virulence plasmid, *Klebsiella pneumoniae*, iron, capsule

## Abstract

Hypervirulent *Klebsiella pneumoniae* causes liver abscess and potentially devastating metastatic complications. The majority of *Klebsiella*-induced liver abscess are caused by the CG23-I sublineage of hypervirulent *Klebsiella pneumoniae*. This and some other lineages possess a >200-kb virulence plasmid. We discovered a novel protein IroP nestled in the virulence plasmid-encoded salmochelin operon that cross-regulates and suppresses the promoter activity of chromosomal type 3 fimbriae (T3F) gene transcription. IroP is itself repressed by iron through the ferric uptake regulator. Iron-rich conditions increase T3F and suppress capsule mucoviscosity, leading to biofilm formation and cell adhesion. Conversely, iron-poor conditions cause a transcriptional switch to hypermucoid capsule production and T3F repression. The likely acquisition of *iroP* on mobile genetic elements and successful adaptive integration into the genetic circuitry of a major lineage of hypervirulent *K. pneumoniae* reveal a powerful example of plasmid chromosomal cross talk that confers an evolutionary advantage. Our discovery also addresses the conundrum of how the hypermucoid capsule that impedes adhesion could be regulated to facilitate biofilm formation and colonization. The acquired ability of the bacteria to alternate between a state favoring dissemination and one that favors colonization in response to iron availability through transcriptional regulation offers novel insights into the evolutionary success of this pathogen.

**IMPORTANCE:**

Hypervirulent *Klebsiella pneumoniae* contributes to the majority of monomicrobial-induced liver abscess infections that can lead to several other metastatic complications. The large virulence plasmid is highly stable in major lineages, suggesting that it provides survival benefits. We discovered a protein IroP encoded on the virulence plasmid that suppresses expression of the type 3 fimbriae. IroP itself is regulated by iron, and we showed that iron regulates hypermucoid capsule production while inversely regulating type 3 fimbriae expression through IroP. The acquisition and integration of this inverse transcriptional switch between fimbriae and capsule mucoviscosity shows an evolved sophisticated plasmid-chromosomal cross talk that changes the behavior of hypervirulent *K. pneumoniae* in response to a key nutrient that could contribute to the evolutionary success of this pathogen.

## INTRODUCTION

Bacteria can be endowed with new-found virulence traits through acquisition of plasmids. Enteric pathogens such as *Salmonella enterica*, *Shigella,* and enteropathogenic *Escherichia coli* have acquired virulence plasmids that are stably associated with the hosts. These virulence plasmids are generally large (>40 kb) and present in single or low copy numbers (1). *Klebsiella pneumoniae* is a member of the *Enterobacteriaceae* family but is not known to possess such virulence plasmids. However, a new hypervirulent breed, that emerged in Taiwan since the 1980s (2) and began spreading sporadically worldwide (3, 4), has acquired a large virulence plasmid that greatly enhances its virulence (5, 6). Hypervirulent *K. pneumoniae* can infect healthy individuals and is mostly known as the primary cause of monomicrobial *Klebsiella*-induced liver abscess (KLA) in Asia (7). However, it can systemically disseminate in the infected host causing pneumonia and endophthalmitis (2).

The rapid expansion and global dissemination of the hypervirulent *K. pneumoniae* CG23-I sublineage that first emerged in 1928 accounted for >80% of KLA infections (8). These strains possess a >200-kb low copy number large virulence plasmid (*Kp*VP). Many hypervirulent *K. pneumoniae* biomarkers such as the *rmpADC* operon that controls hypermucoid capsule production (9, 10)*,* aerobactin (*iuc*) and salmochelin (*iro*) iron-sequestering siderophores (11), and a putative membrane transporter (12) are encoded on the *Kp*VP. It possesses its own plasmid maintenance systems and post-segregational killing of plasmid-lacking cells through toxin-antitoxin systems (1). Interestingly, *Kp*VPs do not carry native antibiotic resistance genes or conjugation machinery but are highly stable in hypervirulent *K. pneumoniae*. This suggests that it provides a benefit to these strains and could contribute to the stability and global dissemination of the CG23-I sublineage.

*K. pneumoniae* thrive ubiquitously in environments from soil and water to animals (13). To survive in harsh conditions, bacteria form communities in biofilms after establishment on certain surfaces (14). Key factors that facilitate this include the capsule and surface adhesions such as the type 1 and 3 fimbriae (15, 16). As hypervirulent *K. pneumoniae* infections in patients are often thought to originate from the gut, it is postulated that the bacteria are acquired from contaminated food sources and translocated through the gut into the bloodstream before systemic dissemination. In fact, CG23 hypervirulent *K. pneumoniae* was found on cucumber samples in China (17). However, its environmental reservoirs are undefined.

Current understanding of hypervirulent *K. pneumoniae*’s ability to acclimatize in environmental habitats and human hosts is still lacking. While the *Kp*VP was reported to be a major virulence factor in mice during intraperitoneal infection (18), its role in the bacterium’s ability to thrive in extracellular habitats or other host niches is unknown. The basis of *Kp*VP’s contribution to mammalian virulence is attributed to the hypermucoid capsule and the major siderophores. However, the extent of their contribution is variable and sometimes contradictory, depending on the genetic profile of the isolates (19, 20). Although the capsule is known to be important for systemic virulence (21, 22), it is not clear how hypermucoid capsule is regulated in *K. pneumoniae* during infection such as to allow colonization, as the mucoviscosity of the capsule is known to impede cell adhesion (23) and yet shown to be important for virulence in a mouse pneumonia model (9).

In this study, we describe the discovery of a novel regulator designated IroP on the *Kp*VP that represses the type 3 fimbriae (T3F). IroP’s expression is inhibited by the presence of iron through the ferric uptake regulator (Fur). We propose that the acquisition of this genetic switch in CG23 K1 lineage allows inverse regulation of T3F and hypermucoid capsule production in a synchronized manner, allowing alternation between a hypermucoid phenotype with low T3F and a less mucoviscous capsule with high T3F phenotype to adapt to changing environments. This versatility could represent a powerful survival tactic for the dominance of the CG23-I. Our finding also addresses the conundrum of how hypervirulent *K. pneumoniae* regulates its capsule and fimbriae in relation to the environment to suit different stages of its life cycle.

## RESULTS

### The loss of *Kp*VP upregulates T3F transcription

**Fig 1:**
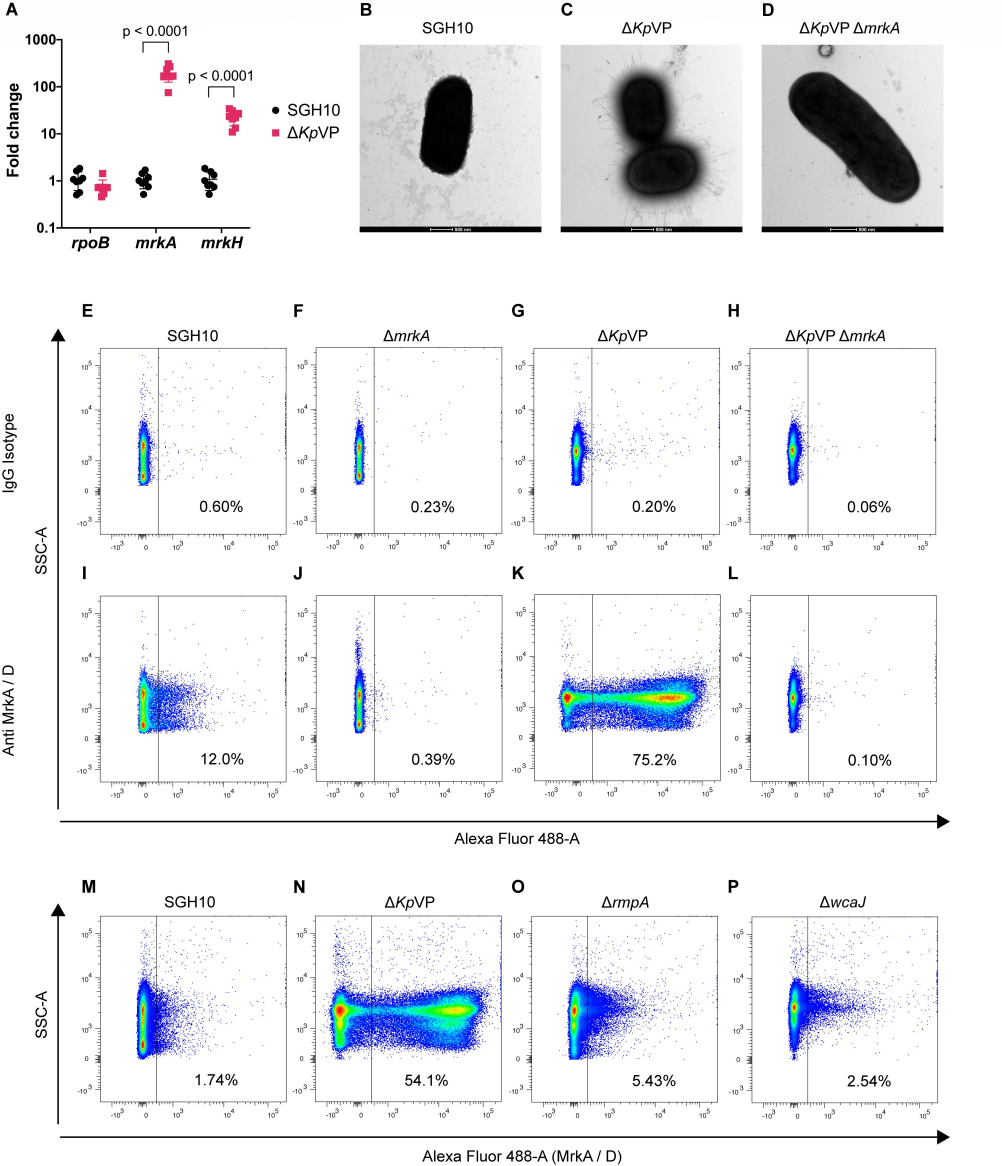
Type 3 fimbrial transcriptional expression and surface protein expression in SGH10 and mutants. (A) RT-qPCR analysis of *mrkA* and *mrkH* transcripts in SGH10 and SGH10 Δ*Kp*VP. Four experiments were conducted with biological duplicates, with mean and error bars representing standard deviation. Unpaired Student’s *t*-test with statistically significant *P*-values (*P* < 0.05) is shown. (B–D) TEM images of SGH10 (B), SGH10 Δ*Kp*VP (C), and SGH10 Δ*Kp*VP Δ*mrkA* (D). Samples for SGH10 and SGH10 Δ*Kp*VP were taken at 11,000×, whereas SGH10 Δ*Kp*VP Δ*mrkA* was taken at 13,000×. Scale bar represents 500 nm. (E–L) Representative flow cytometry plots to detect the T3F. Plots represent IgG isotype controls of SGH10 (E), SGH10 Δ*mrkA* (F), SGH10 Δ*Kp*VP (G), SGH10 Δ*Kp*VP Δ*mrkA* (H), rabbit anti-MrkA/D polyclonal antibody staining of SGH10 (I), SGH10 Δ*mrkA* (J), SGH10 Δ*Kp*VP (K), and SGH10 Δ*Kp*VP Δ*mrkA* (L). Side-scatter area (SSC-A) showing bacterial cells was plotted against Alexa-488 fluorescence. (M*–*P) Representative flow cytometry plots of T3F levels in SGH10 (M), SGH10 Δ*Kp*VP (N), SGH10 Δ*rmpA* (O), and SGH10 Δ*wcaJ* (P).

We examined global changes to the bacterial transcriptome after curing *Kp*VP to determine how *Kp*VP can potentially contribute to changes in bacterial phenotypes. We replaced the VagD toxin in the *Kp*VP major toxin-antitoxin system of CG23-I prototypical strain SGH10, with a rhamnose-inducible RelE toxin cassette (24) to cure the plasmid. RNA-Seq analysis revealed that the 10 most upregulated genes included the T3F encoding *mrk* cluster (Table S1). RT-qPCR validation showed a significantly higher *mrkA* (major structural subunit) and *mrkH* (T3F transcriptional regulator) expression in SGH10 Δ*Kp*VP (Fig. 1A). Transmission electron microscopy (TEM) imaging of SGH10 Δ*Kp*VP showed surface filaments that were absent in SGH10 and SGH10 Δ*Kp*VP Δ*mrkA* (Fig. 1B through D). Flow cytometry validated the higher surface T3F protein expression in SGH10 Δ*Kp*VP (Fig. 1E through L). This demonstrates that the *Kp*VP encodes a T3F suppressive factor. As the capsule could conceal surface adhesions such as the type 1 fimbriae (15, 23), we examined whether the thick hypermucoid capsule could also be masking T3F detection. Both the non-hypermucoviscous SGH10 Δ*rmpA* mutant and the capsule-null SGH10 Δ*wcaJ* mutant showed T3F levels comparable with SGH10. In contrast, only SGH10 Δ*Kp*VP exhibits high levels of T3F (Fig. 1M through P). This clarifies that the capsule is not masking detection and does not block the binding of the anti-MrkA/D antibody to T3F.

### An uncharacterized open reading frame suppresses T3F expression

**Fig 2:**
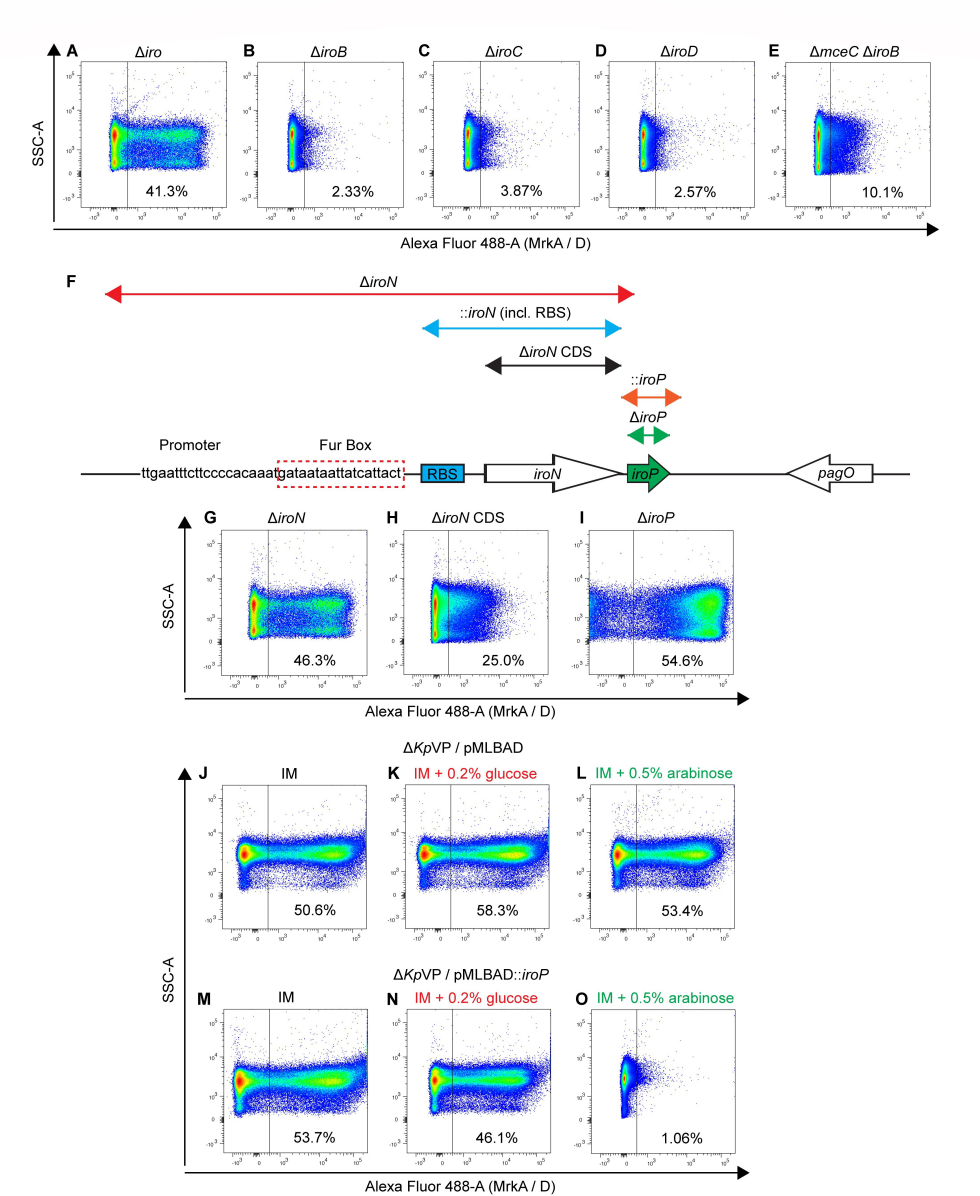
Salmochelin cluster (*iro*) and a novel open reading frame (ORF) *iroP* in the regulation of T3F. (A–E) Representative flow cytometry plots of T3F in SGH10 *iro* deletion mutants, SGH10 Δ*iro* whole cluster deletion (A), SGH10 Δ*iroB* (B), SGH10 Δ*iroC* (C), SGH10 Δ*iroD* (D), and SGH10 Δ*mceC* Δ*iroB* (E). (F) Schematic of the salmochelin *iroN* operon, showing the deleted regions in mutants as well as the regions of gene complementation. The novel ORF, *iroP,* is indicated in green. (G–I) Representative flow cytometry plots of T3F levels in SGH10 Δ*iroN* (G), SGH10 Δ*iroN* CDS (H), and SGH10 Δ*iroP* (I). (J–O) Representative flow cytometry plots of SGH10 Δ*Kp*VP/pMLBAD::*iroP* comparing with SGH10 Δ*Kp*VP/pMLBAD as negative control. SGH10 Δ*Kp*VP/pMLBAD grown in normal infection medium (IM) (J), IM + 0.2% glucose (K), or IM + 0.5% arabinose (L) and SGH10 Δ*Kp*VP/pMLBAD::*iroP* grown in IM (M), IM + 0.2% glucose (N), or IM + 0.5% arabinose (O) are shown. SSC-A, side scatter area.

To identify the *Kp*VP encoded T3F-repressive factor, we first deleted the region encompassing aerobactin (*iuc*), *rmpADC*, and salmochelin (*iro*) operons (SGH10 Δ*iuc-rmp-iro*). The locations of the *iuc*, *rmp*, and *iro* clusters are shown in Fig. S1A. Higher detection of T3F was observed in SGH10 Δ*iuc-rmp-iro* than SGH10 and was comparable with SGH10 Δ*Kp*VP (Fig. S1B and C). Deletion of *iucC* (aerobactin synthetase) and *iucD* (acetyltransferase) (25) significantly reduced siderophore production as shown by a quantifiable CAS assay but did not derepress T3F (Fig. S1D and E). However, deletion of the entire *iro* cluster derepressed the T3F (Fig. 2A).

Salmochelin is formed from enterobactin by the action of a C-glucosyltransferase *iroB* on enterobactin (26). It is secreted through a periplasmic transport protein *iroC* (27). Uptake of iron-bound salmochelin is achieved through the receptor *iroN* (28), and iron is then released when the esterase *iroD* degrades salmochelin (29). We constructed single-deletion mutants of each gene and also deleted both *iroB* and its chromosomal homolog *mceC* (30, 31). We validated the elimination of salmochelin synthesis by determining if the strains made functional microcin E492 (mccE492) that requires salmochelin to kill *E. coli*, as it is a siderophore microcin (31, 32). Indeed, SGH10 Δ*mceC* Δ*iroB* no longer kills *E. coli* (Fig. S2A). However, SGH10 Δ*iroB*, Δ*iroC*, Δ*iroD,* and Δ*mceC* Δ*iroB* do not derepress T3F (Fig. 2B through E). This shows that repression of T3F is independent of salmochelin biosynthesis.

Deletion of the *iroN* operon comprising both the promoter and the coding sequence (CDS) (SGH10 Δ*iroN*) derepressed T3F expression (Fig. 2F and G). However, complementation of the *iroN* coding sequence (SGH10 Δ*iroN*/pMLBAD::*iroN* CDS) did not restore repression (Fig. S2B through F), and deletion of the coding sequence (SGH10 Δ*iroN* CDS) did not fully derepress T3F to the same extent as the *iroN* operon deleted mutant (Fig. 2H). We found a 222-bp open reading frame (ORF) located 61 bp downstream of *iroN* which we named *iroP*. This ORF *iroP* is strongly controlled by the *iroN* promoter (Fig. S3A). Deletion of *iroP* upregulated T3F levels comparable with SGH10 Δ*iroN* (Fig. 2I). Complementation of *iroP* on pMLBAD in the Δ*iroP* mutant restored T3F suppression (Fig. S3B through G). Complementation of *iroP* in SGH10 Δ*Kp*VP similarly restored T3F suppression (Fig. 2J through O). This demonstrates IroP as the major, if not the only, T3F-suppressive factor on *Kp*VP.

### Fur represses IroP and capsule mucoviscosity in the presence of iron

**Fig 3:**
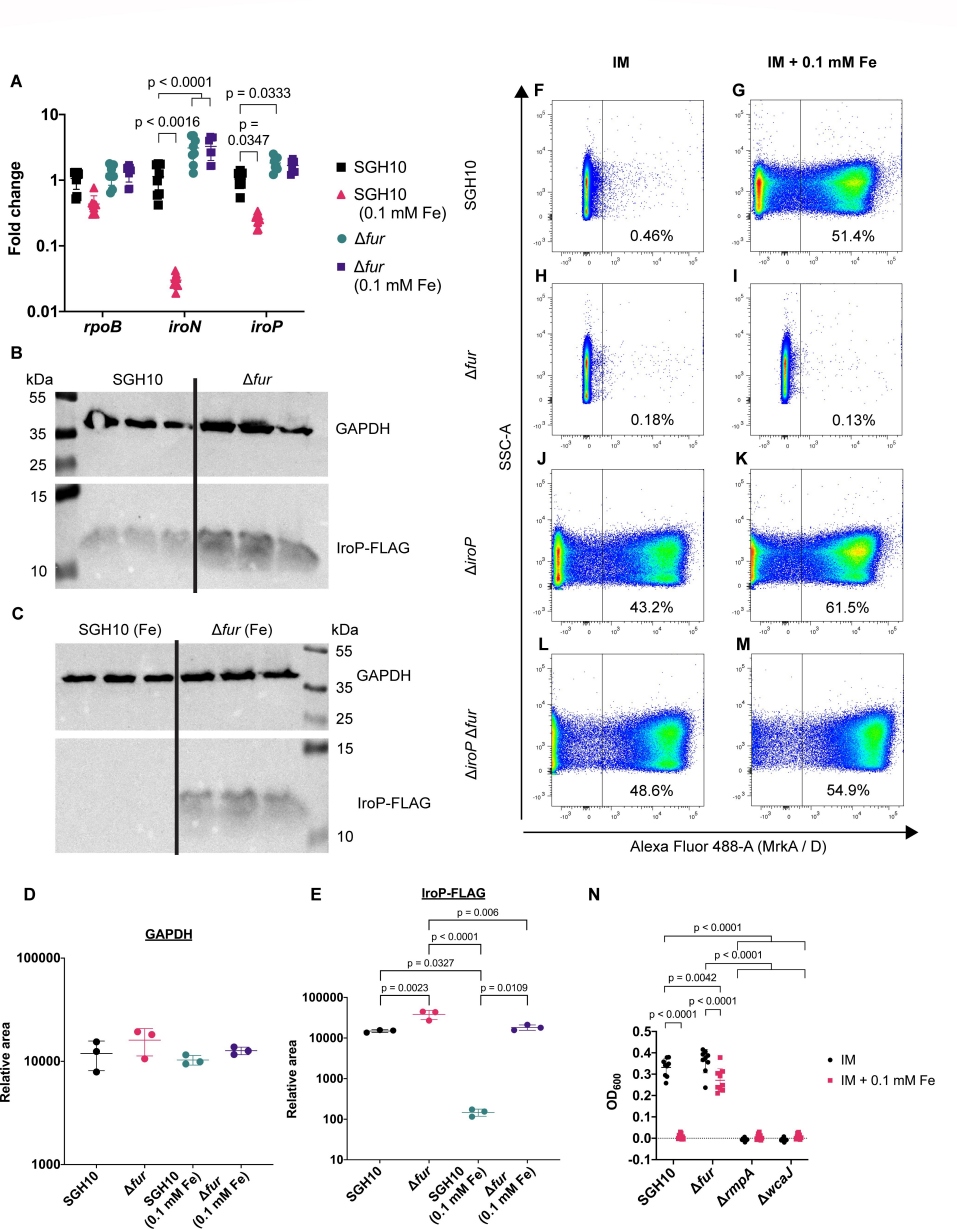
Role of Fur in *iroP* protein expression, T3F regulation, and hypermucoid capsule production. (A) RT-qPCR analysis of *iroN* and *iroP* transcripts in SGH10 and ∆*fur* grown under normal infection medium (IM) or IM + 0.1 mM FeCl_3_. Four experiments were conducted with biological duplicates. Bars represent mean, and error bars represent standard deviation (SD). Gene expressions among the samples were compared using one-way analysis of variance (ANOVA), with *P*-values displayed above the plots in comparison to SGH10. (B) Western blot image of biological triplicate samples of SGH10::*iroP*-FLAG (first three lanes) and SGH10 Δ*fur::iroP*-FLAG (last three lanes) grown in IM. (C) Western blot of biological triplicate samples of SGH10::*iroP*-FLAG (first three wells) and SGH10 ∆*fur::iroP*-FLAG (last three lanes) grown in IM + 0.1 mM FeCl_3_. (D) GAPDH band intensity and (E) IroP-FLAG band intensity from SGH10::*iroP*-FLAG and SGH10 Δ*fur::iroP*-FLAG were quantified using ImageJ. Statistically significant *P*-values (*P* < 0.05) were shown. (F–M) Representative flow cytometry plots of T3F levels, grown under IM or IM + 0.1 mM FeCl_3_ for SGH10 (F and G), SGH10 Δ*fur* (H and I), SGH10 Δ*iroP* (J and K), and SGH10 Δ*iroP* Δ*fur* (L and M). (N) Low-speed centrifugation of SGH10, SGH10 Δ*fur*, SGH10 Δ*rmpA,* and SGH10 Δ*wcaJ* comparing OD_600_ of supernatant of culture grown under normal IM or IM + 0.1 mM FeCl_3_. Three experiments with biological triplicates were conducted, with bars representing mean with SD. Two-way ANOVA statistical analysis was done, and statistically significant *P*-values (*P* < 0.05) were shown. SSC-A, side scatter area.

Although Fur has been described as a transcriptional repressor, it has also been reported to positively regulate the *mrkHI* T3F regulatory loci (33, 34). The salmochelin receptor IroN was shown to be repressed by Fur (35). Since *iroP* is controlled by the P*_iroN_* promoter, we examined the role of Fur in the regulation of T3F via *iroP*. We observed constitutive transcriptional expression of both *iroN* and *iroP* at the beginning of log phase of growth in SGH10 Δ*fur* (Fig. 3A), validating Fur’s role in suppressing *iroN* expression. We tagged a 1× FLAG sequence to native IroP in the *Kp*VP to examine protein expression. Although IroP-FLAG was expressed in both SGH10 and SGH10 Δ*fur*, we observed a significantly lower expression of IroP-FLAG in SGH10 during iron-rich growth (Fig. 3B through E). This translates to a constitutively suppressed T3F in SGH10 Δ*fur* even under iron-rich conditions; however, T3F levels are significantly upregulated in SGH10 under iron-rich conditions. When *iroP* is deleted, there is constitutively high expression of T3F regardless of iron availability (Fig. 3F through M). This shows that Fur acts upstream of IroP in regulating T3F.

Incidentally, the *rmpADC* operon is also regulated by Fur through a Fur binding box at the *rmpADC* promoter (36). Thus, we revalidated the role of Fur with regard to capsule overexpression and mucoviscosity mediated by the *rmp* operon. Although SGH10 could still produce capsule when grown under iron-rich conditions (Fig. S4A through C), mucoviscosity decreased in SGH10 to levels comparable to SGH10 Δ*rmpA* and SGH10 Δ*wcaJ* controls. Deletion of *fur* resulted in a persistent mucoid phenotype regardless of iron concentrations (Fig. 3N). This validates Fur repression of *rmp*-mediated capsule mucoviscosity phenotype under iron-rich conditions.

### Inverse regulation of T3F and hypermucoidy on bacterial phenotypes

**Fig 4:**
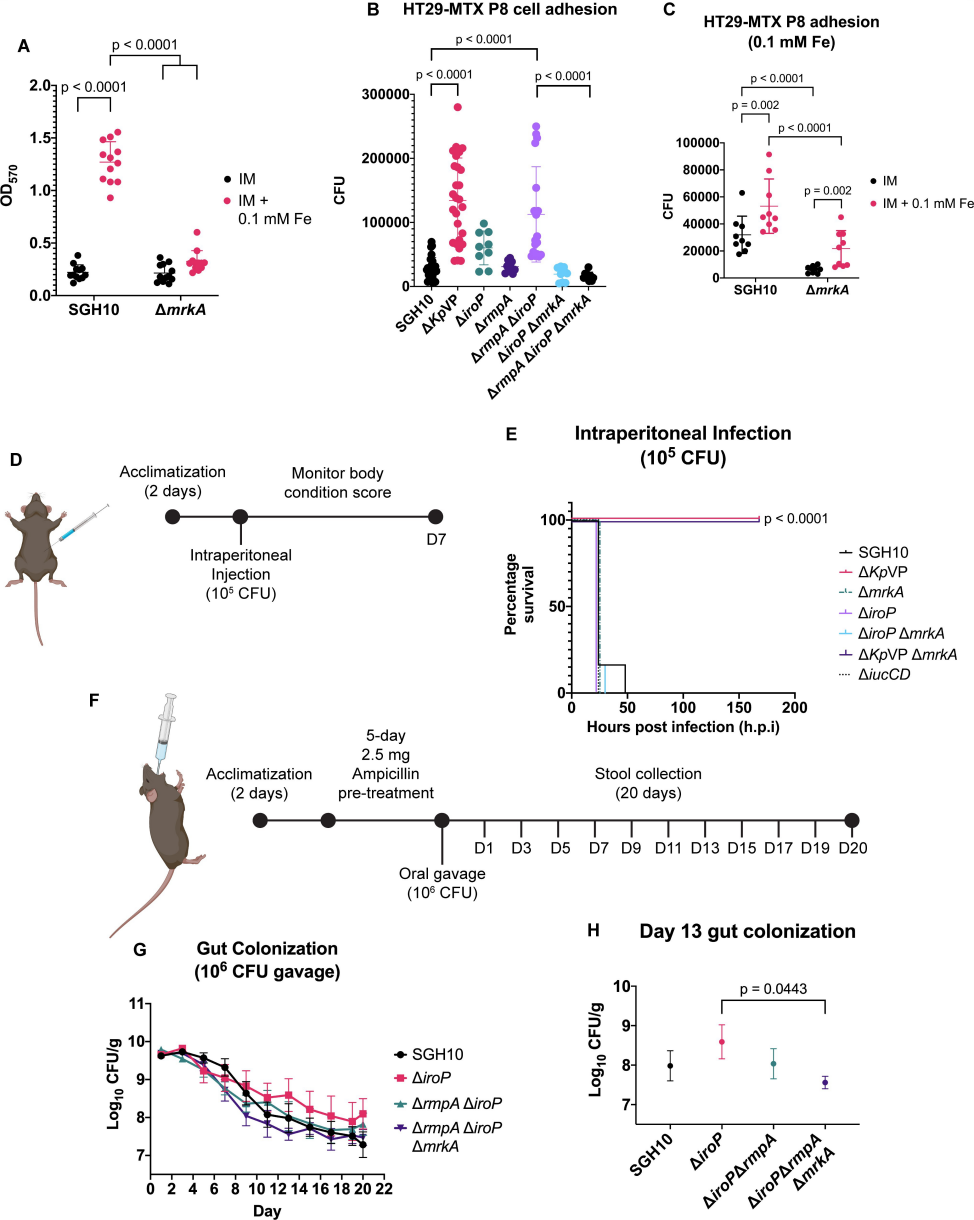
Capsule mucoviscosity and T3F regulation in cell adhesion, biofilm formation, and i*n vivo* infection. (A) Measurement of SGH10 and SGH10 Δ*mrkA* biofilm formation, after growing in normal infection medium (IM) or IM + 0.1 mM FeCl_3_ for 24 h. Four experiments with biological triplicates were conducted. (B) Epithelial cell adhesion of SGH10, SGH10 Δ*Kp*VP, SGH10 Δ*iroP*, SGH10 Δ*rmpA*, SGH10 Δ*rmpA* Δ*iroP*, SGH10 Δ*iroP* Δ*mrkA,* and SGH10 Δ*rmpA* Δ*iroP* Δ*mrkA*. Three experiments with biological triplicates were conducted. (C) Epithelial cell adhesion of SGH10 and SGH10 Δ*mrkA* after growing on IM + 0.1 mM FeCl_3_ iron-rich condition. Three experiments with biological triplicates were conducted. For all the above, bars represent mean with SD. One-way ANOVA analysis was performed, with significant *P*-values indicated above plots in comparison to SGH10. (D) Schematic of mouse intraperitoneal infection drawn using Biorender. (E) Survival curves of mice after 7 d post infection (d.p.i.) with SGH10, SGH10 Δ*Kp*VP, SGH10 Δ*mrkA*, SGH10 Δ*Kp*VP Δ*mrkA*, SGH10 Δ*iroP*, SGH10 Δ*iroP* Δ*mrkA,* or SGH10 Δ*iucCD*, with six mice per group. Mantel-Cox log rank test was conducted. (F) Schematic of C57BL/6 gut colonization of SGH10 and mutant strains via oral gavage. (G) CFU/g of SGH10, SGH10 Δ*iroP*, SGH10 Δ*rmpA* Δ*iroP,* or SGH10 Δ*rmpA* Δ*iroP* Δ*mrkA* in stools after oral infection (in logarithmatic scale). Stools were collected every 2 d until 20 d post infection. (H) CFU/g of SGH10, SGH10 Δ*iroP*, SGH10 Δ*rmpA* Δ*iroP,* or SGH10 Δ*rmpA* Δ*iroP* Δ*mrkA* in stools on day 13 post infection. For G and H, each symbol represents the mean of seven mice. Error bars represent standard deviation. Student’s *t*-test was performed on two groups at a time, with significant *P*-values (*P* < 0.05) as indicated.

Hypervirulent *K. pneumoniae* infection often originated within the patient’s gut (37, 38). The capsule hinders colonic epithelial cell adhesion but does not affect gut colonization (21). As the T3F is also involved in biofilm formation (16, 39), we tested biofilm formation on abiotic surfaces and observed significantly higher biofilm formation by SGH10 under iron-rich conditions facilitated by the T3F (Fig. 4A). Under iron-rich growth, we observed a significantly higher adhesion of SGH10 to HT29 MTX-P8 colon epithelial cells, which was diminished when *mrkA* was deleted (Fig. 4B). These validate the importance of iron regulating the T3F to facilitate both biofilm and cell adhesion. Next, we investigated the role of IroP and hypermucoid capsule production in cell adhesion by infecting HT29 MTX-P8 cells with SGH10 versus various mutants. We observed significantly higher adhesion of SGH10 Δ*Kp*VP to HT29 MTX-P8 cells. The lack of hypermucoid capsule mediated by *rmpA* did not increase adhesion to HT29 MTX-P8 colonic epithelial cells. Deletion of *iroP* slightly increased adhesion despite expressing high T3F, whereas the *rmpA* and *iroP* double mutant showed the highest adhesion comparable to the SGH10 Δ*Kp*VP. Adhesion was attenuated in the Δ*rmpA* Δ*iroP* Δ*mrkA* triple mutant (Fig. 4C). This demonstrates that the hypermucoid capsule is impeding the T3F in colonic epithelial cell adhesion. Thus, the simultaneous suppression of the hypermucoid capsule and derepression of T3F can promote SGH10 binding onto different surfaces.

### IroP regulation on bacterial virulence and gut colonization

We investigated whether IroP and its regulation of T3F play any role in bacterial virulence in a systemic model. By 48 h post infection, all mice succumbed to an infection dose at 10^5^ CFU except for mice infected with SGH10 Δ*Kp*VP or SGH10 Δ*Kp*VP Δ*mrkA* (Fig. 4D and E). This shows that *KpVP* is the major factor affecting systemic virulence. Aerobactin as encoded by the *iuc* operon on *KpVP* had been shown to be important in subcutaneous and intraperitoneal infection in mice (40). However, our aerobactin-deficient Δ*iucCD* mutant was as virulent as SGH10. The loss of virulence in *KpVP* is also not due to upregulation of T3F as the IroP mutant as well as deletion of *mrkA* has no impact on virulence. Our previous work showed that the *rmpA*-null mutant still demonstrated virulence in murine systemic infection (21). Thus, other factors on *KpVP* contribute to systemic virulence.

Since T3F was reported to be important in cellular adhesion (16, 41), we tested single-strain gut colonization in C57BL/6 mice via oral gavage. SGH10-infected mice showed progressive decline in gut colonization by 20 d.p.i. There is a trend where the *iroP* mutant showed higher colonization than SGH10, the double mutant, and the triple mutant, but this did not reach statistical significance when all the groups were compared with one another (Fig. 4F and G). The lack of an obvious effect with the *rmpA* mutation either suggests that hypermucoid capsule is irrelevant for gut colonization or that *rmpA* is already downregulated in SGH10 when in the gut. During day 13, when we compared by Student’s *t*-test the difference between SGH10 Δ*iroP* and the *iroP*, *rmpA,* and *mrkA* triple mutant, we saw a small but statistically significant lower load in SGH10 Δ*iroP* Δ*rmpA* Δ*mrkA* triple mutant (Fig. 4H). Therefore, T3F is not an important contributor to gut colonization in this model.

### IroP prevalence across *K. pneumoniae* isolates

**Fig 5:**
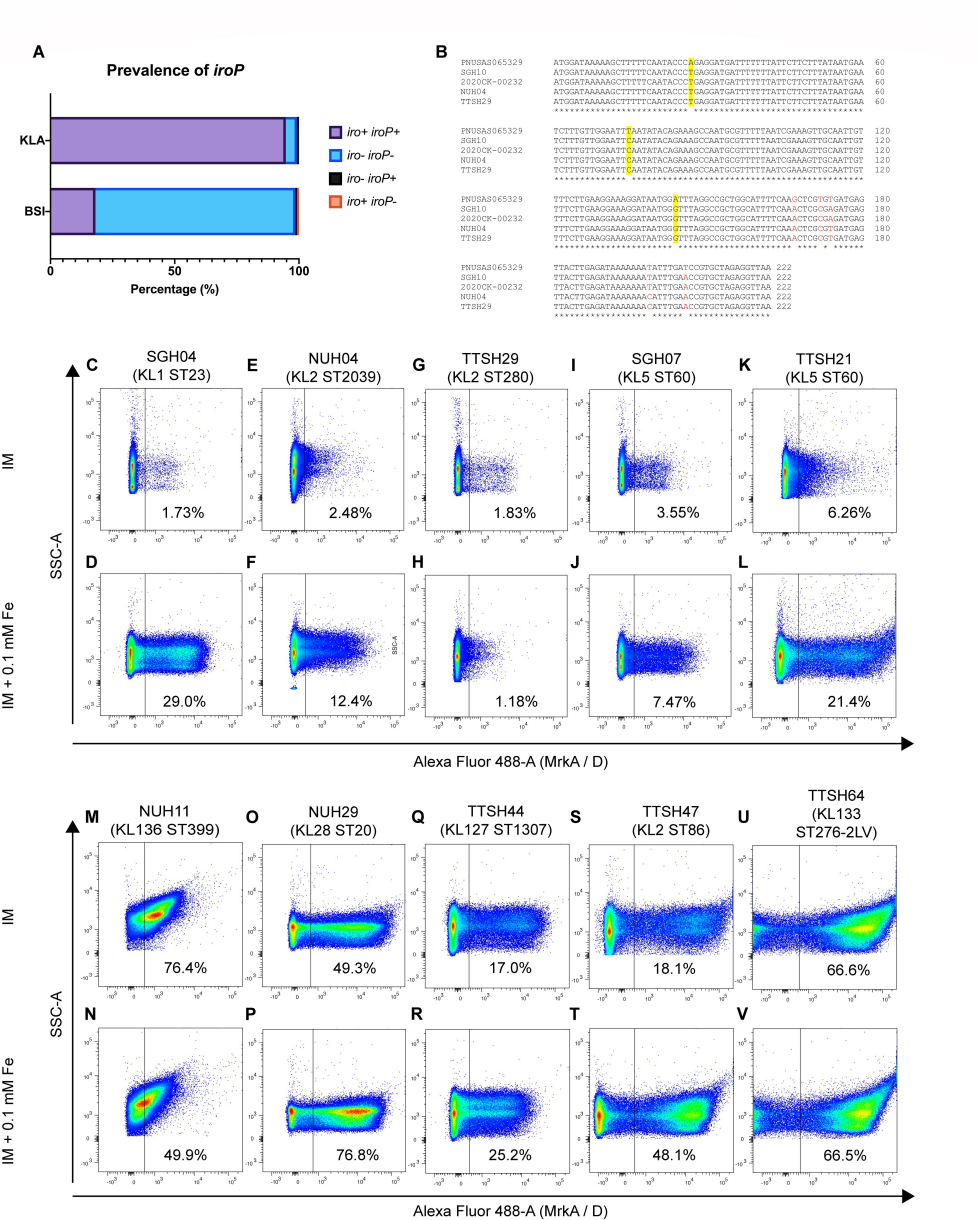
Prevalence of *iroP* and T3F expression in other *K. pneumoniae* isolates. (A) Frequency of *iroP* in strains from KLA or blood isolates from Asian hospitals (BSI) displayed in percentage (%) out of the total number of isolates. (B) Nucleotide sequences of *iroP* in SGH10 were aligned using Clustal Omega to *S. enterica*, *E. coli* urine isolate, NUH04 K2 *Kp*VP, and TTSH29 K2 *Kp*VP. Synonymous mutations are highlighted in yellow, mutations that result in amino acid changes are indicated in red. (C–L) Representative flow cytometry plots comparing T3F levels of selected KLA strains that possess *iroP*. SGH04 possesses *iro1* and was grown under infection medium (IM) (C) or IM + 0.1 mM FeCl_3_ (D). Two strains that possess *iro2* NUH04 grown under IM (E) or IM + 0.1 mM FeCl_3_ (F), and TTSH29 grown under IM (G) or IM + 0.1 mM FeCl_3_ (H). Two strains that possess *iro3* are SGH07 grown under IM (I) or IM + 0.1 mM FeCl_3_ (J), and TTSH21 grown under IM (K) or IM + 0.1 mM FeCl_3_ (L). (M–V) Representative flow cytometry plots comparing T3F levels of *iroP*-negative strains. Plots shown are NUH11 grown in normal IM or IM + 0.1 mM FeCl_3_ (M and N), NUH29 (O and P), TTSH44 (Q and R), TTSH47 (S and T), and TTSH64 (U and V). SSC-A, side scatter area.

In *K. pneumoniae, iro* can be categorized into three allelic variants. K1 capsular strains with pK2044-like virulence plasmids have the *iro1* locus, K2 strains with Kp52.145pII-like virulence plasmids have the *iro2* locus, and strains possessing the chromosomal ICE*Kp*1 mobile element have the *iro3* locus (42). We surveyed the presence of *iroP* in other *K. pneumoniae* strains across 133 KLA isolates (43) and a collection of 365 *Klebsiella* bloodstream infection (BSI) isolates (44). A total of 89 out of the 133 KLA isolates and 27 out of the 365 BSI isolates are of KL1 capsular type. Except for two BSI isolates, all KL1 isolates possess a complete *iro1* locus (Table S2). On the other hand, 22 KLA isolates and 25 BSI isolates are of KL2 capsular type. However, 1 KLA KL2 isolate and 11 BSI KL2 isolates do not have any *iro* clusters. Thirteen KLA KL2 isolates and 11 BSI KL2 isolates possess *iro1* locus, 7 KLA KL2 isolates and 1 BSI KL2 isolate possess *iro2* locus, while 2 BSI KL2 isolates have a truncated *iro3* loci (Table S2). Nevertheless, other capsular subtypes can also possess iro, such as three KLA KL5 isolates that possess *iro1* and two KLA KL5 isolates that possess *iro3* (Table S5). Identical or highly similar *iroP* sequences with at least 95% identity were found across all strains that have the *iro* cluster. However, two KLA strains were found to have a truncated *iroP*, with one having a 170-bp *iroP* with 100% identity to *iroP* in SGH10 and the other having 206 bp but 95% identity to SGH10’s *iroP* (Fig. 5A; Table S5). Prevalence of *iroP* is much lower in the BSI collection. However, strains with the *iro* cluster also possess highly similar or identical *iroP*, with one strain having a truncated *iroP* of 181 bp but 100% identity to SGH10’s *iroP* sequence (Fig. 5A; Table S2). There are some rare exceptions of strains having *iroP* but no *iro* and vice versa—there is one KLA isolate with *iroP* but no detectable *iro* cluster, whereas there are two BSI isolates with *iro* cluster but no *iroP*, and three BSI isolates having *iroP* but not the *iro* cluster.

Our search for the complete sequence of *iroP* in bacteria other than *Klebsiella* species turns out to be limited (Fig. 5B). The *iro* cluster together with *iroP* was found in the chromosome of *Salmonella enterica* isolate PNUSAS065329 (accession number AACZPG010000001), as well as in an unnamed plasmid in a UTI *E. coli* strain 2020CK-00232 (accession number CP107259). IroP in these strains is annotated as hypothetical proteins. Among the KLA isolates, the K2 strains NUH04 and TTSH29 both possess *iroP* on their *Kp*VPs. Nucleotide sequence variations were seen among the various species; however, mutations along the N-terminal end of the protein are synonymous, while missense mutations were found toward the C-terminal region (Fig. 5B). These results suggest hypervirulent *K. pneumoniae* co-acquired *iroP* together with *iro* via mobile genetic elements.

We then compared T3F expression in several KLA strains; SGH04 possesses *iro1* just like SGH10, NUH04 and TTSH29 possess *iro2,* and SGH07 and TTSH21 possess *iro3*. Interestingly, T3F expressions varied across these strains. High iron increased T3F levels of *iro1*-containing SGH04 in a similar trend as SGH10 (Fig. 5C and D). NUH04 (*iro2*) increased T3F levels under iron-rich growth (Fig. 5E and F), whereas T3F expression was undetectable in TTSH29 (*iro2*) even when grown under iron-rich conditions (Fig. 5G and H). SGH07 (*iro3*) showed barely an increase in T3F levels under iron-rich conditions (Fig. 5I and J), whereas TTSH21 (*iro3*) increased from 6.26% to 21.4% (Fig. 5K and L). Five KLA strains that do not possess *iroP* generally have higher T3F levels detected compared to those with *iroP* (Fig. 5M through V). Under iron-rich growth, three of the strains show further increase in T3F. This shows that iron regulation of *iroP* or T3F is not conserved across different lineages and strains.

### IroP suppression of T3F in *K. pneumoniae*

**Fig 6:**
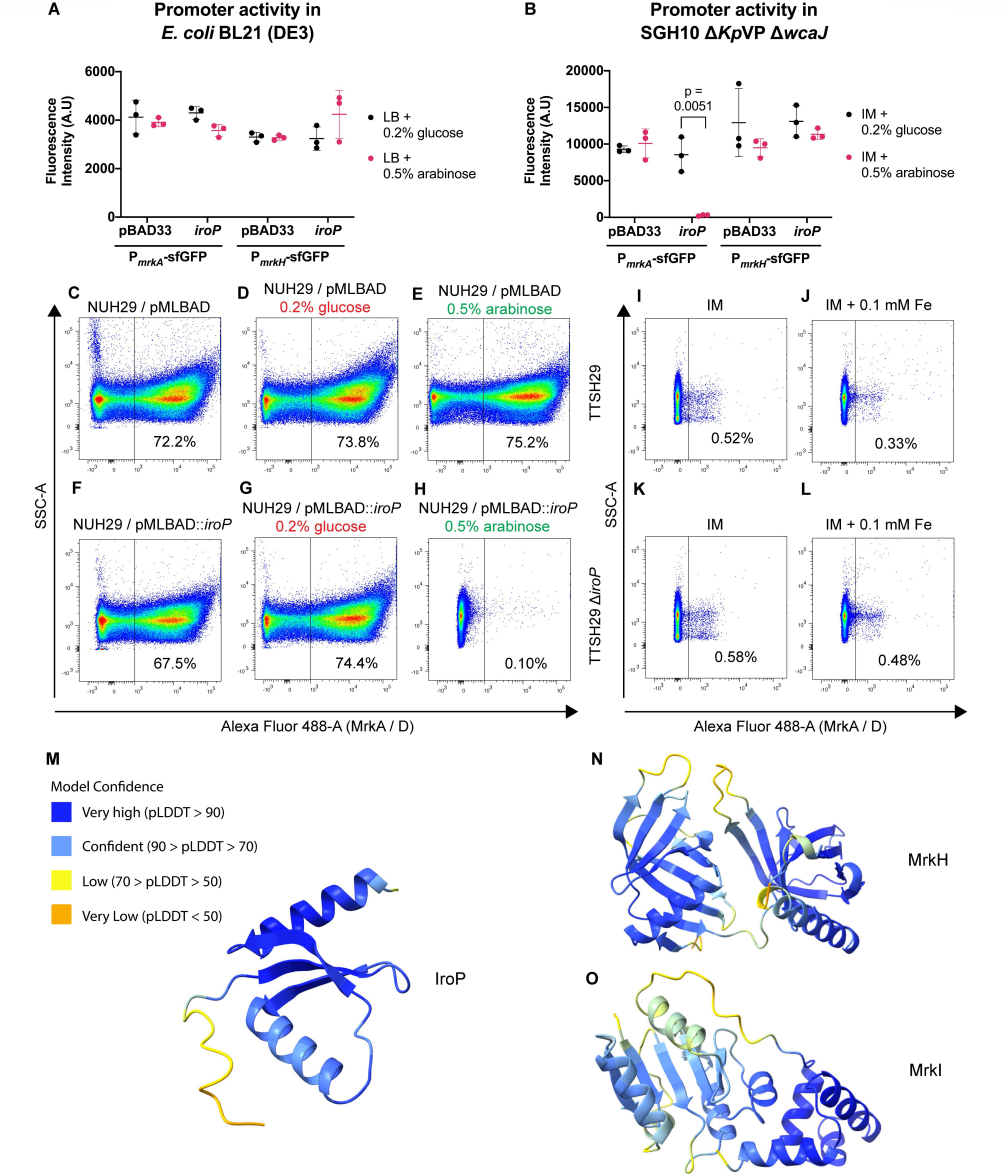
IroP downregulates promoter activity of *mrkABCDF* operon in *K. pneumoniae.* (A) sfGFP reporter assay of P*_mrkA_* or P*_mrkH_* promoters in *E. coli* BL21 (DE3). (B) sfGFP reporter assay of P*_mrkA_* or P*_mrkH_* promoters in SGH10 ∆*Kp*VP ∆*wcaJ*. Each dot represents one colony, with bar representing mean and SD. Significant *P*-values are indicated. (C–H) Representative flow cytometry plots of T3F levels after introduction of *iroP* into NUH29 grown under normal infection medium (IM) (C), IM + 0.2% glucose (D), or IM + 0.5% arabinose (E) as negative control to compare with NUH29/pMLBAD::*iroP* grown under IM (F), IM + 0.2% glucose (G), or IM + 0.5% arabinose (H). (I–L) Representative flow cytometry plots comparing T3F levels of TTSH29 grown in IM (I) or IM + 0.1 mM FeCl_3_ (J) and TTSH29 Δ*iroP* grown in IM (K) or IM + 0.1 mM FeCl_3_ (L). (M–O) Protein structures of SGH10 IroP (M), MrkH (N), and MrkI (O) were predicted using AlphaFold. The model was color coded based on the confidence level of each residue (the predicted local distance difference test [pLDDT] score). Residues with the highest pLDDT scores (>90) were shaded in dark blue, scores between 70 and 90 were shaded in lighter blue, low confidence scores between 50 and 70 were shaded in yellow, and those with the lowest confidence scores (<50) were shaded in orange.

We then examined whether the suppression of T3F could be due to IroP’s suppression of the promoter activity of the *mrkABCDF* and *mrkHI* operons. We utilized *mrkABCDF* and *mrkHI* promoter (P*_mrkA_* and P*_mrkH_*) fusions to superfolder green fluorescence protein (sfGFP) reporters. In *E. coli*, the induction of *iroP* did not significantly decrease sfGFP fluorescence of both P*_mrkA_*-sfGFP and P*_mrkH_*-sfGFP (Fig. 6A). When tested in the SGH10 Δ*Kp*VP Δ*wcaJ* mutant, only P*_mrkA_* but not P*_mrkH_* was suppressed by *iroP* (Fig. 6B). This suggests that *iroP* alone may not be sufficient to act on the promoters and likely requires other co-factors found in *K. pneumoniae* but not in this lab strain of *E. coli*. A capsule-null mutant background was used as the absence of hypermucoid capsule allows reliable recovery of bacteria via centrifugation as well as prevent possible interference of sfGFP fluorescence signals.

As our previous experiments with IroP were done in the CG23-I lineage SGH10, we examine whether the sufficiency of IroP in suppressing T3F is seen in other lineages. NUH29 is a non-hypervirulent *K. pneumoniae* strain without the *Kp*VP or other hypervirulence-associated factors (45, 46). It shows high T3F expression without addition of iron. The exogenous introduction of *iroP* from SGH10 suppressed T3F in NUH29 grown under normal IM (Fig. 6C through H) as well as in the K5 *iro3* strain TTSH21 when grown under iron-rich conditions (Fig. S5A through G). This shows the adequacy of IroP for T3F suppression in *K. pneumoniae* in strains that do not have the *iroP* gene or those with a different *iro* locus. However, when *iroP* was deleted in a K2 hypervirulent strain TTSH29 with negligible T3F expression, T3F was still undetectable (Fig. 6I through L). This points to heterogeneity in IroP regulation.

We further utilized AlphaFold (47, 48) to generate a protein structure prediction of IroP. The predicted structure of IroP is visually represented using color coding based on the predicted local distance difference test (pLDDT) score. The pLDDT scores (Fig. 6M; Fig. S6A) indicated a high level of confidence in the predicted structure. Moreover, the predicted alignment error (PAE) score, which measures the positional error of amino acid residues in angströms (48), demonstrated a low error rate in the domain positions (Fig. S6B). Prediction using DeepFRI (49) on the structure reveals a nucleic acid-binding function with a significant score of 0.58. IroP also has a different protein folding in comparison with the predicted structures of MrkH (Fig. 6N) and MrkI (Fig. 6O). This is further supported by protein structure alignment using TM-align (50), which showed a low score of <0.5 when IroP was compared with both MrkH and MrkI, representing protein folding dissimilarity. The difference in protein structure between IroP and MrkH/MrkI suggests that IroP likely interacts with DNA in a different way from MrkH/MrkI.

## DISCUSSION

Plasmids can benefit bacterial hosts by gaining antibiotic or toxin resistance. They can also affect metabolism, nutrient transport, or virulence gene expressions. For example, the plasmid p24835-NDM5 can upregulate carbohydrate metabolic genes in hypervirulent *K. pneumoniae* (51), whereas pAB04-1 and pAB3 suppress the type VI secretion system in *Acinetobacter baumannii* (52, 53). In *Rhodococcus equi*, plasmid-encoded transcriptional regulators modify the expression of several chromosomally encoded virulence-associated traits to enhance survival of both the bacterium and the plasmid in macrophages (54). These examples are evidence that plasmid-encoded regulators may have important fitness consequences for the plasmid and the host cell (55). We have much to understand about the role of *Kp*VP in *K. pneumoniae* fitness and virulence. Although some *K. pneumoniae* strains have chromosomally incorporated *iuc*, *iro,* and *rmpA* (56, 57), a majority of K1 and K2 hypervirulent *K. pneumoniae* retain the *Kp*VP. Apart from the known *rmp* operon and siderophores, *Kp*VP contains many other genes of unknown functions. Changes in transcriptomic profile due to *Kp*VP loss provide strong indications of cross talk between *Kp*VP and the chromosome, and could reveal regulatory pathways to circumvent fitness costs to the bacterial host.

Interestingly, the biggest change we observed with the loss of *Kp*VP is the upregulation of T3F in the bacteria. T3F is an extended surface appendage found on *Enterobacteriaceae* and known to be an important factor in biofilm formation on abiotic surfaces and during *in vitro* cell adhesion by binding to collagen V (58, 59). The main structure consists of a major subunit MrkA with an adhesion tip MrkD and is assembled through an MrkB chaperone and an MrkC chaperone usher protein (60, 61). MrkF is likely part of the T3F assembly mechanism with unknown functions (62). The entire mechanism is regulated by iron and c-di-GMP, where the latter controls the activity of the positive transcriptional regulator MrkHI. MrkHI in turn binds to the *mrkABCDF* promoter (63). Fur had previously been postulated to regulate T3F through *mrkHI* and a hypothetical unknown repressor of *mrkABCDF* (34). Our findings that *Kp*VP repression of T3F occurs via IroP support this hypothesis, and we believe IroP is that “hypothetical repressor.” However, what is surprising is that IroP is nestled within the *iro* cluster on the large virulent plasmid. Its co-expression with IroN allows its regulation by Fur and iron. We also demonstrated an inverse functional relationship between adhesions and capsule mucoviscosity in influencing biofilm and adhesion. So far, it is not known how hypermucoviscous *K. pneumoniae* navigate its interaction with the host through adhesion and colonization while having a thick and mucoid capsule. The assumption is that capsule must be regulated to expose fimbriae and adhesions for colonization to take place. This may stem from how both are organized on the bacterial surface as T3F interferes with hydrogen bonding between capsular polysaccharides (64). However, as we have shown, T3F is not constitutively expressed in CG23-I, as this would be futile during stages when capsule mucoviscosity is fully expressed. Therefore, the synchronization of the on-off switch of T3F and the degree of capsule hypermucoviscosity prevent the interference of the hypermucoid capsule with T3F and allow bacterial lifestyle adaptation to a biofilm state, or to establish cellular adhesion and colonization. In *K. pneumoniae* ST258, selection pressure for persistence in the bladder in urinary tract infection was conferred by capsule-null mutations that resulted in increased invasion and biofilm formation in the bladder (65). Conversely, blood infections select for those with mutations in *wzc* that resulted in hypermucoviscous capsule that resists phagocytosis. For SGH10, alternation of these traits associated with each phase of infection could be accomplished by the convenience of the Fur-IroP switch instead of through mutant selection, which would confine the mutants to a particular niche.

High biofilm formation in SGH10 under iron-rich condition is attributed to derepressed T3F expression and downregulated capsule mucoviscosity. Interestingly, T3F overexpression on its own does not significantly improve biofilm formation (64). We propose that adhesion can only be achieved when there is an optimal balance between fimbriae levels and the capsule mucoviscosity, and both are mediated by iron availability. Siderophores can play an important role in iron uptake since it contributes to the majority of iron in *P. aeruginosa* and is important for initial establishment and biofilm formation (66). In UTI89 *E. coli*, siderophore iron uptake promotes the production of metabolites required for biofilm formation (67). *K. pneumoniae* strains also produce more siderophores during the planktonic phase than during the sessile state (68). Hypervirulent *K. pneumoniae* strains produce more siderophores than classical strains and can form biofilms more robustly (69). We postulate that during the planktonic phase, iron is acquired leading to a gradual increase in intracellular iron and formation of Fur-Fe complexes. This will gradually repress hypermucoviscosity and increase T3F expression. At this point, bacteria then switch from the planktonic and likely disseminating phase to a sessile state as it colonizes a niche through surface adhesion and/or biofilm formation. This switch may take place in the host gut, given that diet, host factors, and gut microbiome can cross-regulate iron availability. Generally, free iron is extremely low (10^−24^ M) in humans, and most of the iron is bound to high-affinity host proteins. However, higher concentrations of iron are expected to be in the colon as the majority of iron that is not absorbed in the duodenum ends up there (70). In addition, fecal iron excretion in adults who consumed a diet containing a normal concentration of iron at 6–8 mg/d was about 7.5 mg/d (1.3 × 10^−4^ M/d) (71). It is possible that in the colon, there is enough iron from a normal diet to induce Fur activation in hypervirulent *K. pneumoniae*, resulting in high T3F and low capsule mucoviscosity, thereby favoring colonization.

However, despite the clear contribution of T3F and capsule mucoviscosity during *in vitro* cell adhesion and biofilm experiments, the difference in colonic colonization between bacteria with derepressed T3F and mutants with both derepressed T3F as well as deletion of *rmpA* was not seen. This is likely due to the already repressed *rmpA* expression in the colon. The difference contributed by the SGH10 *iroP* deletion mutant and SGH10 in colonization was also not statistically significant, and this would be expected if *iroP* was already suppressed by Fur in SGH10. There was a small difference between the Δ*iroP* and the Δ*iroP* Δ*rmpA* Δ*mrkA* triple mutant, showing that deletion of T3F results in poorer intestinal colonization at day 13, meaning that T3F would have been upregulated in that environment but its role in intestinal colonization remains limited. It is also conceivable that the advantage of this reversible switch is more evident when bacteria are transiting between different environments not tested in our study. We have not explored other niches, such as in the lung, liver, or in extracellular niches such as soil where iron sources could be abundant, especially in acidic soil (72). It would be interesting to find the relevant environments for this transition in the life cycle of hypervirulent *K. pneumoniae* where the role of IroP is significant in bacterial fitness and survival. Interestingly, capsule’s maintenance has been proposed to be driven by as yet unknown factors outside of the host (73). During *in vitro* evolution experiments in different growth media, mutations in capsule and T3F arose more frequently in capsulated and non-capsulated backgrounds, respectively (74). This could reflect as yet characterized roles of capsule and T3F in the environment outside of the host that may be relevant to the lifestyle switch we proposed.

The different lineages of *iro* loci found in *K. pneumoniae, E. cloacae,* or *E. coli* (42) suggest that the regulation of IroP differs across different *iro* loci. Indeed, T3F expression differs across hypervirulent *K. pneumoniae* sequence types as well as non-hypervirulent *K. pneumoniae* isolates. Furthermore, we do not see T3F upregulation under iron-rich condition in non-CG23-I hypervirulent *K. pneumoniae* that possess IroP as significantly as seen in CG23-I. Deletion of *iroP* also does not necessarily lead to T3F upregulation in strains such as TTSH29. This suggests that *iroP* is regulated differently in these strains from what is seen in the CG23-I sublineage. It is interesting to note that IscR, an iron-sulfur cluster-containing transcriptional regulator, has been documented to control T3F and capsule production through the effect of iron in a K2 *K. pneumoniae* isolate CG43 (75). Apo-IscR activates MrkA transcription, whereas holo-IscR which is formed when IscR binds iron-sulfur cluster represses T3F via the direct repression of MrkHI promoter. Apo-IscR does not directly bind to the *mrkA* or *mrkHI* promoters (75). However, holo-IscR activates capsule synthesis genes (76). This means that under iron-rich conditions, T3F is repressed, whereas capsule synthesis is increased. This regulatory control is in direct contrast to the effect of Fur and IroP. The caveat is that Fur represses IscR (75) during iron-rich conditions and supersedes the effects of IscR. Perhaps only in iron-poor conditions, IscR could be opposing the effect of IroP because apo-IscR activates T3F whereas IroP would be derepressed and suppresses T3F. CG43 also possesses an identical IroP to SGH10. It is possible that these competing levels of regulatory control could lead to heterogeneity in T3F regulation by IroP. In our promoter-sfGFP fusion assays, we did not see IroP exerting repression on the *mrkHI* promoter, although it repressed the *mrkA* promoter. This is somewhat surprising as there was transcriptional upregulation of *mrkHI* in the *KpVP* mutant (Fig. 1A). One limitation of this work is that we performed the promoter-fusion assays in a capsule-null mutant background, and capsule’s absence might have affected the repression on *mrkHI*. Alternatively, IroP mainly acts on the *mrkABCDF* operon, and the increase in *mrkHI* seen in Fig. 1A could be due to factors affected by the absence of *Kp*VP. For example, there may be fewer holo-IscR resulting in an increase in *mrkHI* since the loss of *Kp*VP can reduce iron levels due to the absence of iron uptake systems encoded on the plasmid.

Although we did not find any DNA-binding domains using domain and motif searches in IroP, the new DeepFRI platform based on graph convolutional networks on 3D structures shows a strong prediction of DNA-binding function (49). However, the dissimilarity of IroP protein folding compared with MrkH and MrkI suggested that IroP may bind to DNA differently than MrkH and MrkI. This may not be surprising as IroP suppresses, whereas MrkH and MrkI form a complex to upregulate the *mrkA* operon. Nevertheless, further work would be required to examine whether IroP, with or without cofactors, could interact with the promoter region of the *mrkA* operon to directly mediate repression.

Acquisition of salmochelin in hypervirulent *K. pneumoniae* might have evolutionarily yielded an advantageous adaptation to control T3F expression in relation to iron. IroP may be described as a “genome hitchhiker,” given that its function does not seem aligned to the rest of the genes in the salmochelin operon. While it is first identified in *Salmonella*, the *iro* locus is detected in hypervirulent *K. pneumoniae*, *E. cloacae,* and *E. coli* (42). The packaging of both *iro* and *iroP* genes into mobilizable genetic elements such as plasmids or pathogenicity-associated islands has the potential to accelerate the spread and acquisition of a T3F switch, which can provide pathogens with enhanced pathogenesis. In fact, hybrid virulence plasmids that acquired conjugation machinery have been reported to conjugatively transfer to *E. coli* from hypervirulent *K. pneumoniae* (77). A foodborne *E. coli* pathogenic strain EC1108 was also found to possess a virulence plasmid harboring a similar *iuc-rmp-iro* region as those found in hypervirulent *K. pneumoniae* (78). The recent release of an unnamed plasmid sequence that has the identical sequence of *iroP* as SGH10 in human urine *E. coli* isolate implies that the salmochelin cluster is capable of disseminating across different strains in the urinary tract. This could be of major concern as T3F is a well-known virulence factor during urinary tract infections. Rising reports of mobile elements carrying the *iro* locus could mean that other *Enterobacteriaceae* species apart from *K. pneumoniae* and *E. coli* can also acquire the salmochelin cluster. Interestingly, a recent report documented the wide distribution of *rsm* genes and its homologs on plasmids in diverse taxa (79). This is an example of a plasmid global translational regulator evolved to control the bacterial behavioral switch from a motile to a sessile lifestyle and bacterial metabolism through plasmid chromosomal cross talk. The authors propose that plasmids may commonly control bacterial lifestyle in the clinic, agricultural settings, and other environments.

**Fig 7:**
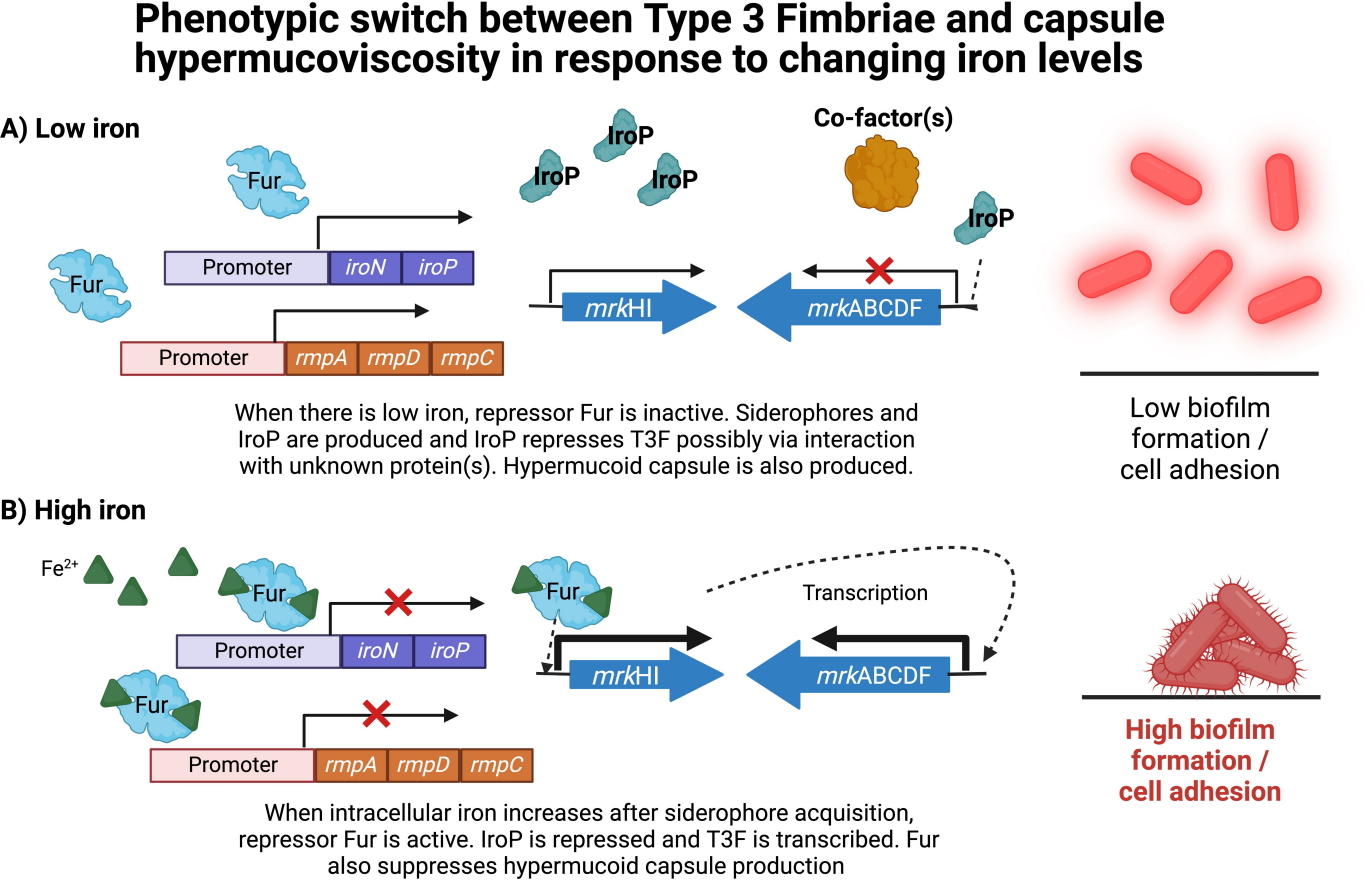
Transcriptional phenotypic switch between T3F and capsule hypermucoviscosity in response to changing iron levels. (A) Low iron condition results in hypermucoid capsule production and repressed T3F, leading to low biofilm formation and cell adhesion. (B) Iron-rich condition results in expression of T3F and downregulation of capule mucoviscosity, leading to high biofilm formation and cell adhesion. Image was created using Biorender.

In conclusion, we discovered a novel regulatory pathway mediated by the Fur-regulated IroP found on the *Kp*VP. IroP likely acts with other transcription factors to regulate the T3F in a plasmid-chromosomal cross talk. We propose that Fur-Fe-mediated regulation of T3F and hypermucoid capsule provides a reversible transcriptional switch for bacteria navigating through different environmental niches with variable iron availability. Iron limitation inactivates Fur, allowing expression of both *rmpADC* and *iroP,* which leads to T3F suppression, in turn impeding biofilm formation while facilitating hypervirulent *K. pneumoniae* dissemination. Iron repletion triggers Fur-Fe binding to the relevant promoters, suppressing *iroP* and *rmpADC* while upregulating *mrkABCDF* leading to high T3F production with low hypermucoid capsule and promoting biofilm formation, epithelial cell adhesion, and perhaps gut colonization (Fig. 7). During systemic circulation where free iron sources are limited, the phenotype of hypermucoid capsule and low T3F dominates to aid in dissemination. This regulatory network allows CG23 hypervirulent *K. pneumoniae* to alternate between a hypermucoid phenotype versus a phenotype with high T3F and a basal level of capsule production, modulating its adhesion to various surfaces in different niches. This presents another powerful example of how plasmid-encoded regulators can become tightly interwoven with chromosomal gene regulation through a complex cross talk that is coupled to a key and valuable nutrient iron. This regulation endows the bacteria with new-found abilities to deftly navigate through harsh conditions that likely contribute to their evolutionary success. Whether this IroP regulatory switch will spread and be integrated into more lineages and species through mobile genetic elements remains to be seen.

## MATERIALS AND METHODS

### Bacterial strains and growth conditions

List of strains used are described in Table S3. All *E. coli* strains were grown at 37°C in Lysogeny Broth (LB) (ThermoFisher Scientific, Waltham, MA, USA), whereas *K. pneumoniae* strains were grown at 37°C in Dulbecco’s modified Eagle’s medium (DMEM) (Invitrogen/ThermoFisher Scientific) + 10% fetal bovine serum (FBS) (Biowest, Nuaille, France), which constitutes the infection media (IM). For microcin diffusion and mouse gut colonization, LB was used.

### Deletion of *Kp*VP in SGH10

Kanamycin resistance and rhamnose-inducible *relE* cassette from pSLC-217 (24) as well as *vagD* upstream and downstream flanking sequences were amplified using Q5 Polymerase (NEB, Ipswich, MA, USA) and assembled in pR6KmobsacB zeo (R6K replication origin replaced pMB1 in pK18mobsacB (80) with zeocin resistance) using the HiFi Assembly Master Mix (NEB). Plasmids were electroporated into S17 *E. coli,* and colonies selected on LB agar with 100 µg/mL kanamycin and 50 µg/mL zeocin. Colonies were mixed at 10 *E. coli* : 1 SGH10 ratio for 24 h and plasmids carrying SGH10 were selected on LB agar plates + 100 µg/mL kanamycin + 100 µg/mL carbenicillin. A chosen colony was then grown for 24 h at 37°C in LB with 20% sucrose and plated on LB agar + 100 µg/mL kanamycin to counterselect for colonies with rhamnose inducible RelE cassette. Selected colonies were subsequently grown in LB with 2% rhamnose to induce RelE and then plated on normal LB agar plates. Cured *Kp*VP mutant strains were screened for seven genes (*parA1*, *copC*, *tRNA*, *coA*, *rmpA*, *terC,* and *dppC*) by qPCR. The list of primers for amplification of *relE* cassette and qPCR primers are listed in Tables S2 and S3, respectively.

### Construction of gene mutants

Gene deletion in SGH10 was performed as previously described (21). In brief, Q5 amplified upstream and downstream regions of the target gene together with pR6KmobsacB were assembled and introduced via conjugation into SGH10 using *E. coli* S17 as donor. Selection was done with 100 µg/mL kanamycin and 100 µg/mL carbenicillin. Negative selection was performed by growing in LB with 20% sucrose and spread on LB agar. Colonies were screened using PCR to validate gene deletion. All primers used are listed in Table S4.

### RNA isolation, cDNA reverse transcription, and quantitative real-time qPCR

Overnight cultures were subcultured into new IM media and grown for 2 h at 37°C. Trizol-chloroform method was performed to isolate RNA using Purezol (Bio-rad, Hercules, CA, USA) and the Purelink RNA Mini Kit (ThermoFisher Scientific). cDNA was synthesized using the Maxima H Minus Synthesis Kit (ThermoFisher Scientific). Real-time qPCR was done using iQ SYBR Green Supermix (Bio-rad). Relative RNA transcripts were normalized against the wild-type control using the 2^-ddCt^ threshold cycle method (81) with the *recA* as the reference gene and *rpoB* as the internal housekeeping control. All qPCR primers are listed in Table S5.

### TEM sample preparation and imaging

OD_600_ of overnight bacterial cultures were measured and diluted to 10^8^ CFU/mL. Cells were pelleted at 2,000 × *g* centrifugation for 7 min and fixed with 2.5% glutaraldehyde in 1× phosphate buffered saline (PBS) (pH 7.3) for 2 h at 4°C. Cells were washed twice with 1× PBS (pH 7.3) for 20 min on ice. Fixed cells were air dried on the transmission electron microscope formvar and carbon-covered grid for 5 min, followed by staining with 1% phosphotungstic acid for 1 min. Images were taken with the help of the electron microscopy unit.

### Flow cytometry

A volume of 10^7^ CFU/mL of overnight culture was stained on ice in the dark for 1 h with anti-MrkA/D rabbit primary antibodies at 1:650 µL ratio. Secondary goat anti-rabbit Alexa-488 conjugated antibodies (ThermoFisher Scientific) were added at 0.5:400 µL ratio in 1 × PBS and incubated on ice in the dark for 40 min. Three hundred microliters of 1% formaldehyde (Sigma, St Louis, MI, USA) were added dropwise to fix cells and incubated at room temperature for 15 min. Formaldehyde was removed, and each sample was resuspended in PBS for analysis. All samples were acquired for 100,000–200,000 events and gated for *K. pneumoniae*. Detection of target population was gated based on the rabbit IgG isotype control of the individual strains. The side-scatter area depicting detection of bacterial cells was plotted against Alexa-488 fluorescence.

### SDS-PAGE and western blot

An amount of 1 mg/mL crude lysate was prepared by boiling colonies obtained from an overnight culture in 1× Laemmli buffer (Bio-rad) with 1 × SDS (Vivantis, Czech Republic). A range of 10–20 μg was loaded to a 17% resolving, 6% stacking polyacrylamide gel and run using Mini-Protean Gel (Bio-rad). Nitrocellulose membrane transfer was done using the Mini-Transblot (Bio-rad). FLAG-tagged proteins were detected using anti-FLAG rabbit polyclonal antibody (Biolegend, San Diego, CA, USA) at 1:750 µL dilution and horseradish peroxidase (HRP)-conjugated anti-rabbit antibody (Immunology Consultants Laboratory, Portland, OR, USA) at 1:3,000 µL. As loading control, bacterial glyceraldehyde dehydrogenase (GAPDH) was detected with anti-GAPDH HRP conjugated antibody (Invitrogen) at 1:2,000 µL. Blot was developed using Pierce ECL Western Blotting Substrate (ThermoFisher Scientific). Band intensity was quantified as peak area using ImageJ.

### Mucoviscosity test using low-speed centrifugation

Low-speed centrifugation test was conducted according to our protocol as previously described (21). In brief, 10^9^ CFU/mL of overnight cultures was spun down at 2,000 × *g* for 10 min. Two hundred microliters of supernatant were aliquoted into a 96-well polystyrene plate, and absorbance was measured at OD_600_.

### Cell culture

A549 was cultured in DMEM supplemented with 10% FBS and 1× penicillin-streptomycin (Biowest). HT29-MTX P8 (82) was cultured in DMEM supplemented with 20% FBS, 1× non-essential-essential amino acids (Gibco/ThermoFisher Scientific) and 1× penicillin-streptomycin. All mammalian cells were maintained in a humidified incubator at 37°C and 5% CO_2_.

### Bacterial cell adhesion

HT29-MTX P8 were seeded at a cell density of 0.25 × 10^6^ on 24 well plates. Bacterial infection of cells was done at multiplicity of infection of 10 bacteria:1 cell and incubated for 30 min at 37°C. After washing thrice in 1× PBS, cells were lysed with 0.1%–0.25% Triton X-100 (Sigma). Appropriate dilutions were plated on lysogeny broth agar (LBA) and incubated at 37°C overnight.

### Biofilm assay

A volume of 10^9^ CFU/mL of overnight culture was seeded in triplicates on a 96-well polystyrene plate and incubated at 37°C for 24 h. Biofilm was washed with 1× PBS, fixed in 100% methanol, and stained in 0.1% crystal violet. Residual crystal violet was washed with ddH_2_O until clear. Crystal violet from stained biofilm was solubilized in 70% ethanol, and absorbance was measured at A_570_.

### Intraperitoneal mouse infection

A volume of 10^5^ CFU in 100 µL of bacterial culture was injected into the intraperitoneal cavity of each mouse. Mice were monitored twice daily until day 7 post infection. Based on the body condition score system (5—healthy active mouse to 1—severely ill, not able to keep upright), mice that fell within body condition score of 2 were euthanized and harvested for organs. Organs were homogenized using 2.8-mm ceramic beads (Omni International, Kennesaw, GA, USA) in 1 × PBS using a Beadrupter (Omni International). Appropriate dilutions were plated on Klebsiella Selective Agar (KSA).

### Gut colonization

Ampicillin pre-treatment of 2.5 mg in 100 µL was administered to each mouse via oral gavage for 5 d. After pre-treatment, all mice were infected via oral gavage with 10^6^ CFU bacteria in 100 µL. Each mouse was individually separated into different cages to prevent coprophagy. Stools from all mice were collected every 2 d post infection and homogenized using 1.4-mm ceramic beads (Omni International) in 1 × PBS. Appropriate dilutions were spread plated on KSA plates.

### Gene alignment using Pathogenwatch and BLAST

The sequence of *iroP* was obtained using the ORFfinder prediction (83). Sequences of the entire *iro* operon as well as *iroP* were queried using Pathogenwatch (84) and aligned using BLAST to databases obtained from the A-KLASS study comprising 133 whole-genome sequences (43) and the collection comprising 365 whole-genome sequences (44), which we refer to as “BSI.” Sequences of A-KLASS KLA isolates NUH11, NUH27, NUH29, SGH04 and SGH07 were previously uploaded to Genbank under Bioproject PRJNA351910 . Sequences of the remaining KLA isolates were uploaded to Genbank under Bioproject PRJNA956314.

### sfGFP promoter activity measurement

Overnight cultures, grown for 24 h, were spun down and diluted to OD_600_ of 0.5 in 1 × PBS. Two hundred microliters of the culture were aliquoted into 96-well black plates with clear bottom (Corning, New York, NY, USA) with technical duplicates. GFP fluorescence intensity was measured at 485 nm emission and 510 nm excitation.

### Protein structure prediction using AlphaFold

IroP protein structure was predicted via AlphaFold (v2.3.1) (47), using Docker on an in-house system. To obtain the pairwise confidence measure, multiple sequence alignments were performed against the full database (db_preset = full_dbs) and model_preset as monomer_ptm. The predicted structure was visualized using ChimeraX (85), while python script was utilized to generate pLDDT and PAE graphs.

### Statistical methods

Student’s *t*-test was used to compare means and standard deviation of two groups. For three groups or more, a one-way or two-way ANOVA with Dunnett’s multiple comparison was performed to compare means and standard deviation relative to the wild type. For mouse colonization model, each group was individually compared with a second group at each time point, using Student’s *t*-test to compare means and standard error of means. Survival curve was analyzed with Mantel-Cox log rank test. All graphical and statistical methods were performed using Prism Graphpad 9 (GraphPad Software, La Jolla, CA, USA).

## Data Availability

All data needed to evaluate the conclusions in the paper are present in the paper and the supplemental material. Sequences of KLA isolates were uploaded to Genbank under Bioproject PRJNA956314.

## AFTER EPUB

[This article was published on 2 August 2023 with errors in the Fig. 4 legend. The legend was corrected in the current version, posted on 7 August 2023.]

## SUPPLEMENTAL MATERIAL

Figure S1 mbio.01297-23-s0001.tif Partial KpVP deletion of *iuc-rmp-iro* region derepresses T3F but is independent of aerobactin.

Figure S2 mbio.01297-23-s0002.tif T3F suppressive phenotype is independent of salmochelin synthesis and uptake.

Figure S3 mbio.01297-23-s0003.tif The novel open reading frame *iroP* represses the T3F and is controlled by the P*_iroN_* promoter.

Figure S4 mbio.01297-23-s0004.tif Iron supplementation unlikely affects capsule production.

Figure S5 mbio.01297-23-s0005.tif Induction of *iroP* in hypervirulent *K. pneumoniae* K5 strain TTSH21 also suppresses T3F expression when grown under iron supplementation.

Figure S6 mbio.01297-23-s0006.tif IroP protein structure was predicted with high confidence using Alphafold.

Supplemental text mbio.01297-23-s0007.docx Supplemental methods, legends, and Tables S3–S5.

Table S1 mbio.01297-23-s0008.xlsx RNAseq data.

Table S2 mbio.01297-23-s0009.xlsx Pathogenwatch and blast results.

ASM does not own the copyrights to Supplemental Material that may be linked to, or accessed through, an article. The authors have granted ASM a non-exclusive, world-wide license to publish the Supplemental Material files. Please contact the corresponding author directly for reuse.
