# Supplementary figures and images for: Acquisition of regulator on virulence plasmid of hypervirulent Klebsiella allows bacterial lifestyle switch in response to iron
Source: mBio. 2023 Aug 2;14(4):e01297-23. doi: 10.1128/mbio.01297-23 (PMC10470599; doi:10.1128/mbio.01297-23)

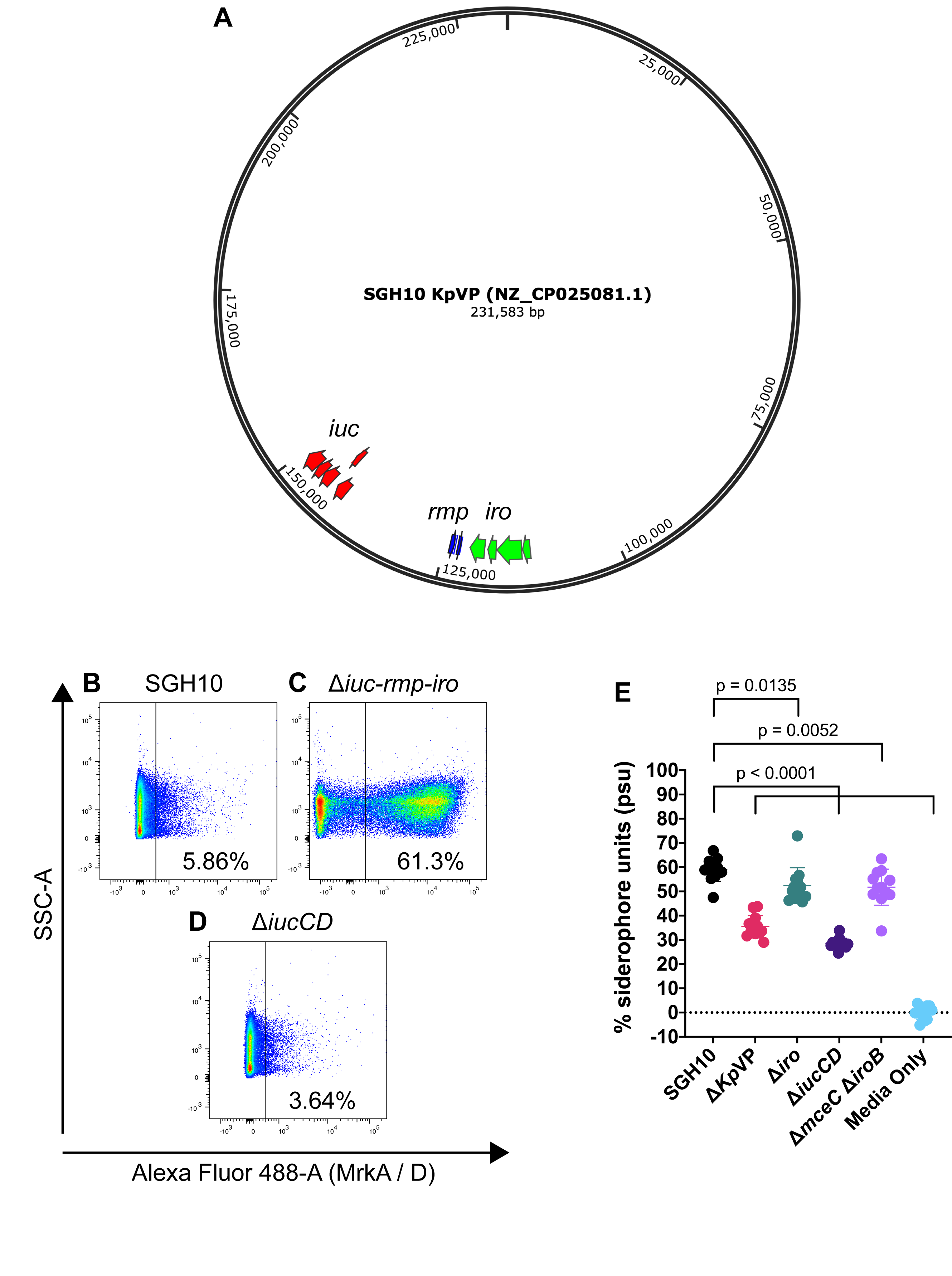

Supplement: Figure S1 — Partial KpVP deletion of iuc-rmp-iro region derepresses T3F but is independent of aerobactin. [file mbio.01297-23-s0001.tif]

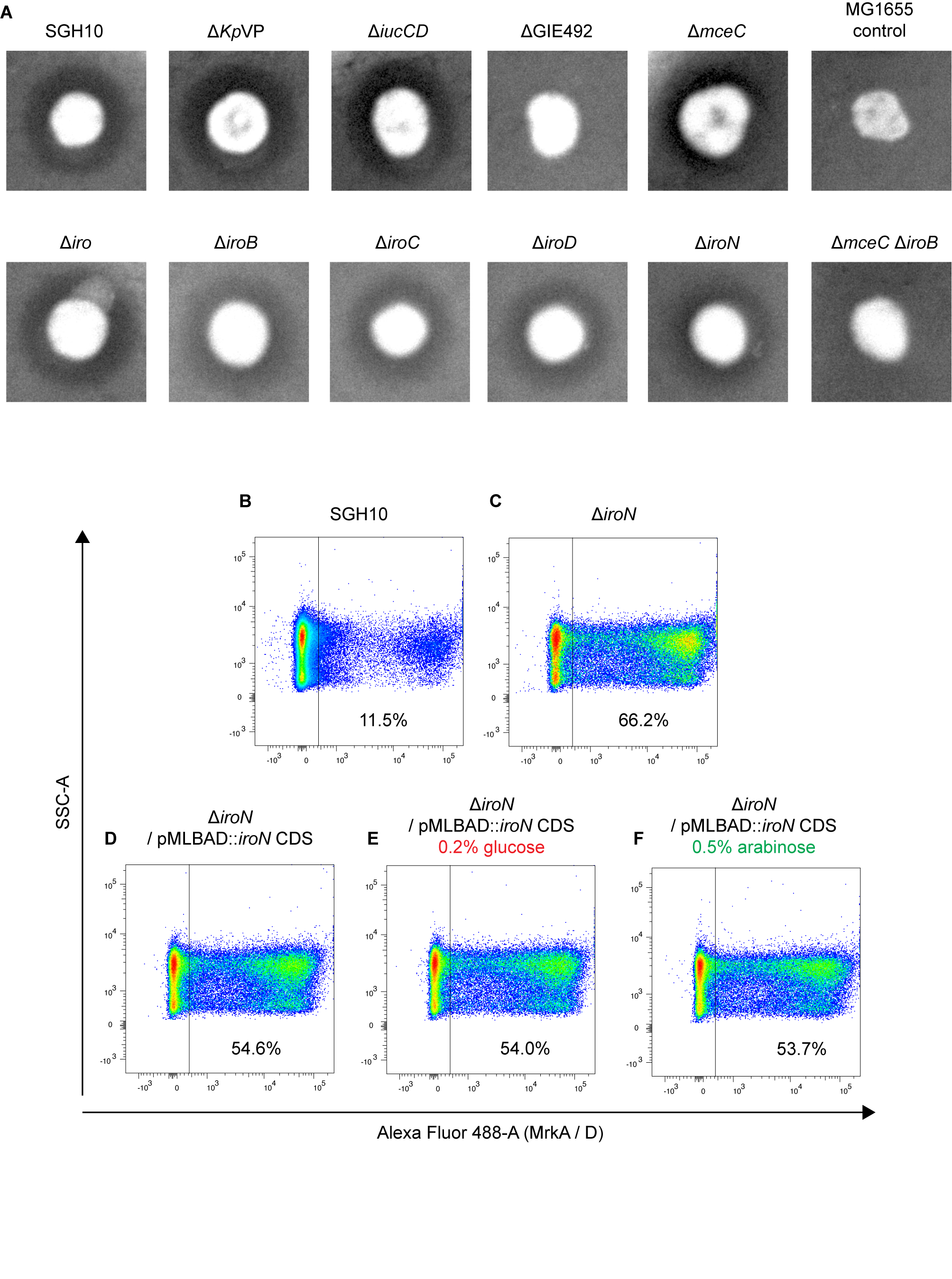

Supplement: Figure S2 — T3F suppressive phenotype is independent of salmochelin synthesis and uptake. [file mbio.01297-23-s0002.tif]

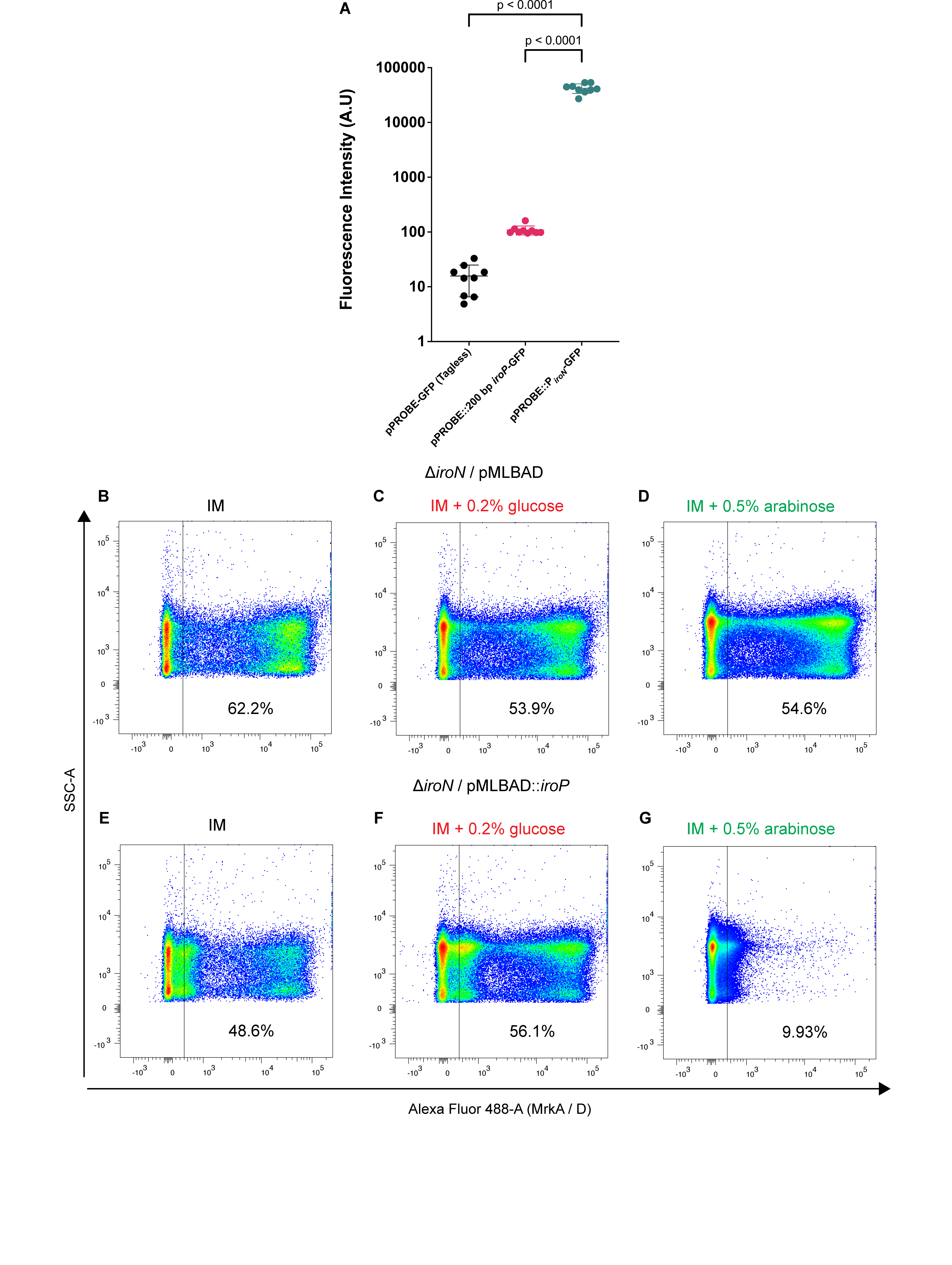

Supplement: Figure S3 — The novel open reading frame iroP represses the T3F and is controlled by the PiroN promoter. [file mbio.01297-23-s0003.tif]

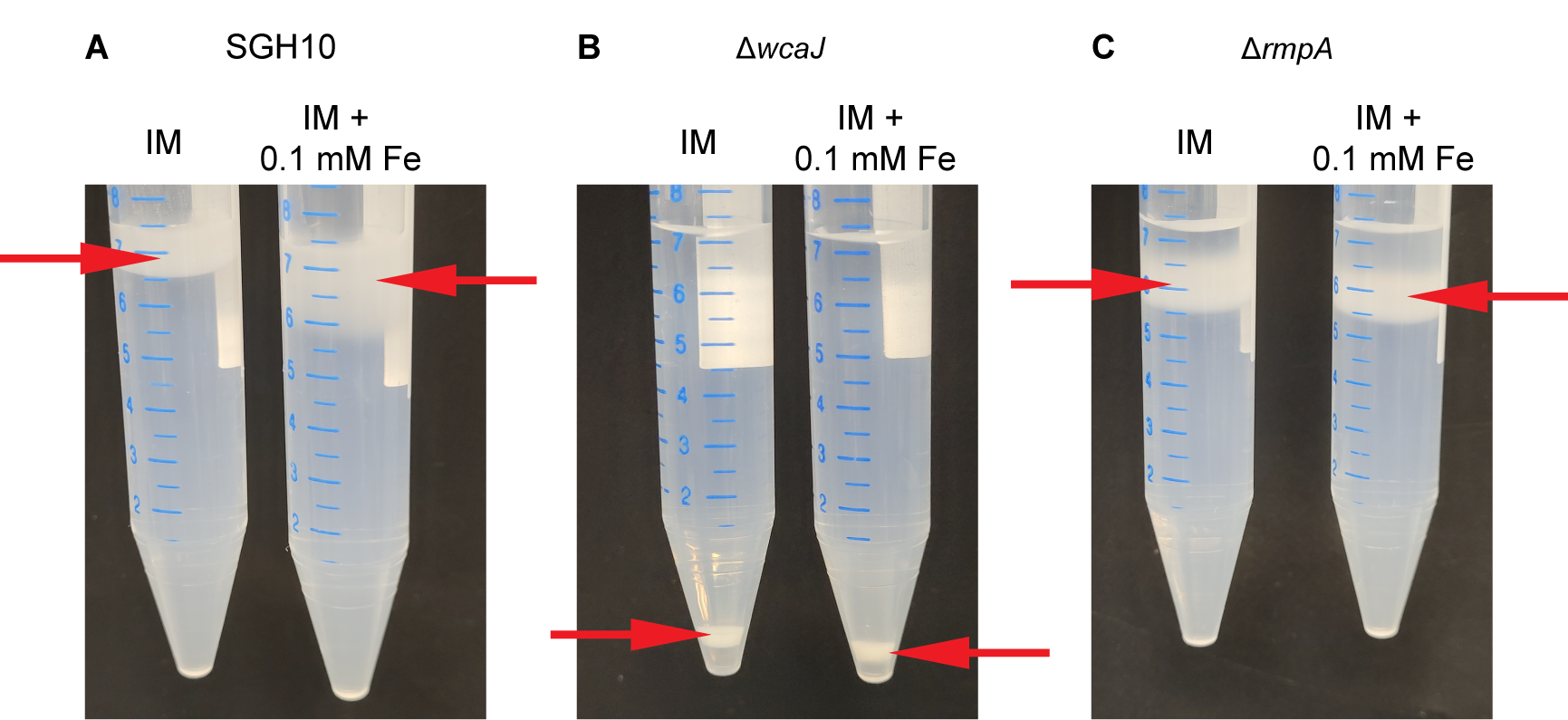

Supplement: Figure S4 — Iron supplementation unlikely affects capsule production. [file mbio.01297-23-s0004.tif]

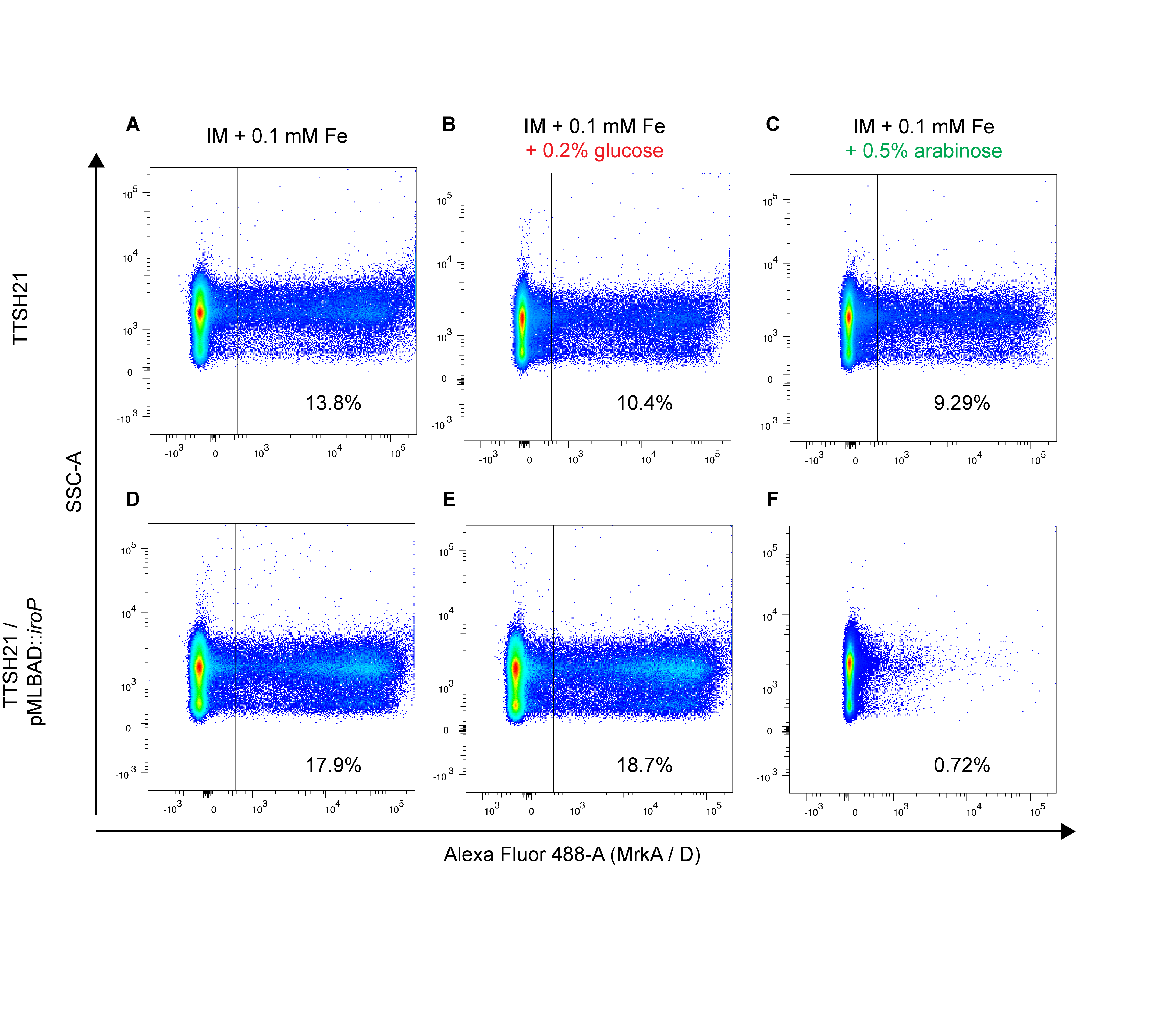

Supplement: Figure S5 — Induction of iroP in hypervirulent K. pneumoniae K5 strain TTSH21 also suppresses T3F expression when grown under iron supplementation. [file mbio.01297-23-s0005.tif]

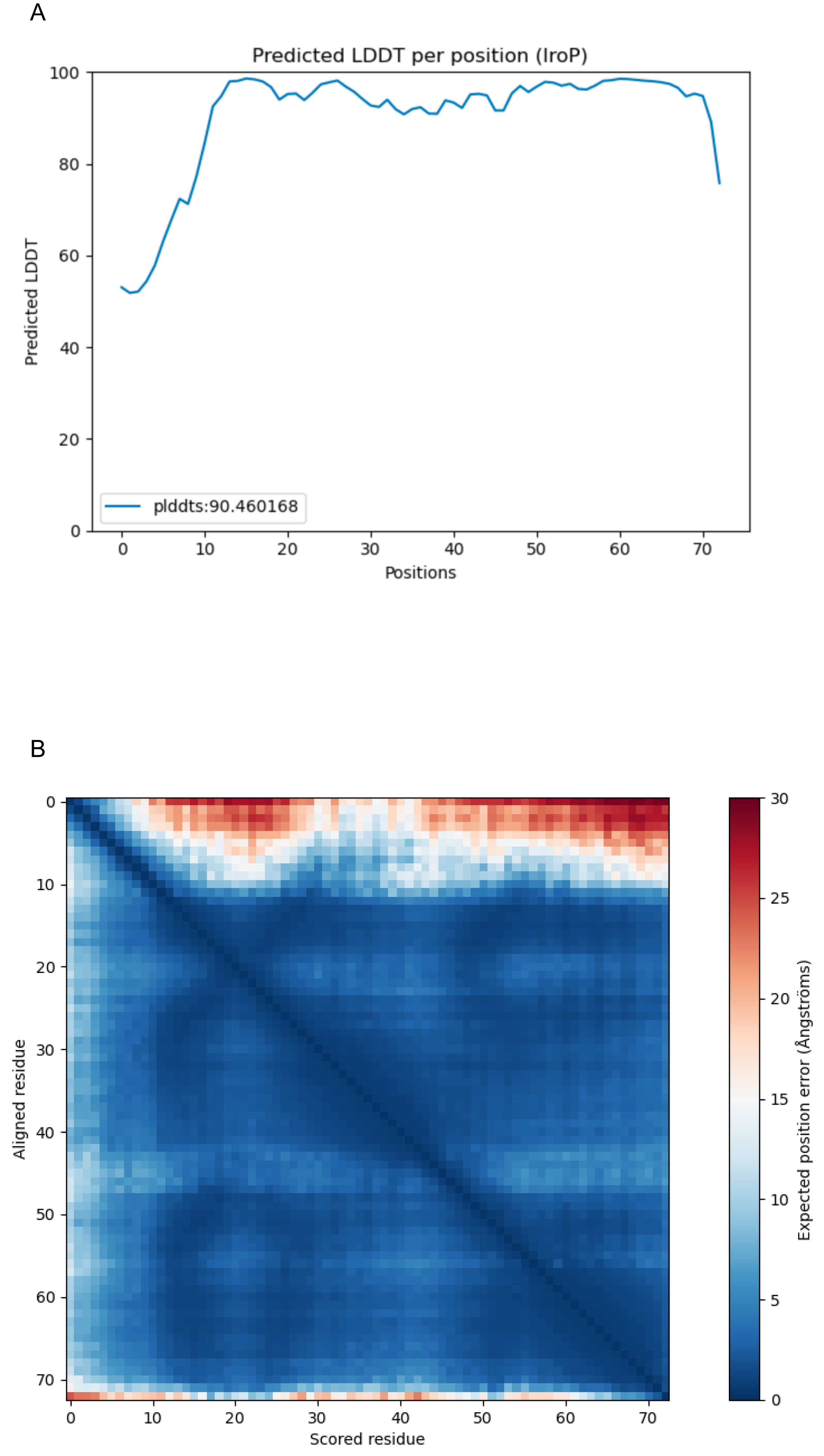

Supplement: Figure S6 — IroP protein structure was predicted with high confidence using Alphafold. [file mbio.01297-23-s0006.tif]
