# Supplementary material for: Acquisition of regulator on virulence plasmid of hypervirulent Klebsiella allows bacterial lifestyle switch in response to iron
Source: mBio. 2023 Aug 2;14(4):e01297-23. doi: 10.1128/mbio.01297-23 (PMC10470599; doi:10.1128/mbio.01297-23)
Supplement: Supplemental text — Supplemental methods, legends, and Tables S3–S5. [file mbio.01297-23-s0007.docx]

Supplementary Materials & Methods

RNAseq analysis

Biological duplicate RNA samples were obtained after samples were pre-treated with 200 μL of pre-heated Trizol^TM^ Max (Thermofisher) based on manufacturer’s recommended protocol. The RNA samples were sent for Illumina HiSeq analysis (Novogene). Gene expression levels were calculated based on the Fragments Per Kilobase of transcript sequence per Millions base pair-sequenced (FPKM) method (1). Normalization was performed using DESeq (2) and negative binomial distribution was conducted to calculate statistical significance.

Chrome Azurol S (CAS) Siderophore Assay

CAS assay was performed following protocol previously described by (3) with modifications. In brief, overnight cultures were subcultured in quadruplicates to 10^8^ CFU/mL and grew at 37 °C for 24 hrs. Samples were spun down at 3000 × *g*, 10 mins and 100 μL of supernatant was mixed with 100 μL of 4× CAS reagent in a 96-well flat bottom clear plate (Corning). After 1 hr incubation at 37 °C, absorbance was measured at OD_630_.

Microcin E492 Diffusion Assay

Experiment was conducted following previous protocols (4-6). In brief, 30 μL of overnight culture of *E. coli* MG1655 prey was diluted in molten 0.35% soft LB agar and poured onto LBA plate. After solidifying, 1 μL of overnight *K. pneumoniae* bacterial cultures were spotted gently onto LBA and left at room temperature (R.T.) for 13 hrs.

Low Density Percoll Gradient

A Percoll gradient assay to determine capsule production was conducted following our previous described protocol (7) with modifications. In brief, 5 × 10^9^ CFU of bacteria was resuspended in 600 μL of 1× PBS. From highest to lowest percentage, 2.0 mL of 50%, 35% and 15% Percoll (SigmaAldrich) in 1× PBS was added to a 15 mL Falcon^®^ tube. Bacteria were resuspended and slowly added into tube and spun at 3000 × *g* for 30 mins. Image of tubes was then taken against a dark background.

**Fig. S1. Partial *Kp*VP deletion of *iuc-rmp-iro* region derepresses T3F but is independent of aerobactin.**

(**A**) Schematic diagram of SGH10 *Kp*VP, with positions of aerobactin cluster *iuc* (in red), regulator of mucoid phenotype cluster *rmp* (in blue) and salmochelin cluster *iro* (in green). Diagram was created using Snapgene. (**B** to **D**) Representative flow cytometry plots of SGH10 (A), SGH10 Δ*iuc-rmp-iro* (B) and SGH10 Δ*iucCD* (C). (**E**) CAS Assay comparing SGH10, SGH10 Δ*Kp*VP, SGH10 Δ*iro*, SGH10 Δ*iucCD* and SGH10 Δ*mceC* Δ*iroB* with media only as negative control. 4X CAS reagent was mixed 1:1 with supernatant and OD630 was measured. Three experiments were performed with biological quadruplicates, each dot representing each replicate. Bar represents mean while scale bar represents standard deviation. Statistical significance was analyzed using one-way ANOVA with statistically significant of p-values (p<0.05) against the wildtype shown.

**Fig. S2. T3F suppressive phenotype is independent of salmochelin synthesis and uptake.**

(**A**) Salmochelin synthesis stops when *mceC* and *iroB* are deleted. Microcin E492 bactericidal activity tested using diffusion assay comparing SGH10 WT, SGH10 Δ*Kp*VP, SGH10 Δ*iro*, SGH10 Δ*iroB*, SGH10 Δ*iroC*, SGH10 Δ*iroD*, SGH10 Δ*iroN*, SGH10 ΔGIE492, SGH10 Δ*mceC* and SGH10 Δ*mceC* Δ*iroB* with *E. coli* MG1655 as prey. Killing of MG1655 was represented as clear halo surrounding *K. pneumoniae*. (**B** to **F**) Representative flow cytometry plots of SGH10 Δ*iroN* / pMLBAD::*iroN* CDS. SGH10 WT (B) and SGH10 Δ*iroN* (C) grown in DMEM + 10% FBS IM normal, and SGH10 WT Δ*iroN* / pMLBAD::*iroN* CDS grown under DMEM + 10% FBS IM (D), IM + 0.2% glucose (E) or IM + 0.5% arabinose (F) are shown. Goat anti-rabbit Alexa-488 conjugated antibody was used as secondary antibody.

**Fig. S3. The novel open reading frame *iroP* represses the T3F and is controlled by the P*_iroN_* promoter.**

(**A**) pPROBE tagless-GFP, pPROBE::200 bp *iroP* upstream and pPROBE::P*_iroN_* were electroporated into SGH10 Δ*wcaJ* and GFP fluorescence intensity after a 24-hr incubation in DMEM + 10% FBS and was measured at 485mm / 510mm emission-excitation wavelength. Three experiments with biological triplicates were done. Bar represents mean while scale bar represents standard deviation. Significant values of p < 0.05 were displayed. (**B** to **G**) Representative flow cytometry plots of T3F levels expressed in SGH10 Δ*iroP* / pMLBAD::*iroP* comparing with SGH10 Δ*iroP* / pMLBAD as negative control. Samples for SGH10 Δ*iroP* / pMLBAD grown in DMEM + 10% FBS IM (B), IM + 0.2% glucose (C) or IM + 0.5% arabinose (D) and for SGH10 Δ*iroP* / pMLBAD::*iroP* grown in DMEM + 10% FBS IM (E), IM + 0.2% glucose (F) or
IM + 0.5% arabinose (G) are shown.

**Fig. S4 Iron supplementation unlikely affects capsule production.**

**(A to C)** Percoll gradient comparing SGH10 WT **(A)**, Δ*rmpA* **(B)** and Δ*wcaJ* **(C)** grown under normal DMEM + 10% FBS IM (left) or IM + 0.1 mM FeCl_3_ (right) of one image representing each strain.

**Fig. S5. Induction of *iroP* in hypervirulent *K. pneumoniae* K5 strain TTSH21 also suppresses T3F expression when grown under iron supplementation.**

(**A** to **F**) Representative flow cytometry plots showing MrkA and MrkD expression in K5 strain TTSH21 after growing in IM + 0.1 mM FeCl_3_ (A), IM + 0.1 mM FeCl_3_ + 0.2% glucose (B) or IM + 0.1 mM FeCl_3_ + 0.5% arabinose (C), compared with MrkA and MrkD levels of TTSH21 / pMLBAD::*iroP* grown under IM + 0.1 mM FeCl_3_ (D), IM + 0.2% glucose + 0.1 mM FeCl_3_ (E) or IM + 0.5% arabinose + 0.1 mM FeCl_3_ (F).

**Fig. S6 IroP protein structure was predicted with high confidence using Alphafold.**

(**A**) Predicted local-distance difference test (pLDDT) depicting confidence was plotted based on amino acid position (x-axis) against the pLDDT score (y-axis). (**B**) Positional error between amino acid residues was calculated as predicted alignment error (PAE) in Ångströms. Low to high error scores were colored from blue to red.

Table S1. (separate file)

**RNAseq FPKM read count results of all upregulated and downregulated genes comparing SGH10 and SGH10 Δ*Kp*VP after 2 hrs inoculation in DMEM + 10% FBS (beginning of log phase).** Values calculated from biological duplicates.

Table S2. (separate file)

**BLAST and Pathogenwatch overview of KLA and BSI genome sequences comparing *iro* and *iroP* sequences with that of SGH10 as query sequence.**

Table S3. Table of bacterial strains used in this study.

| **Strain** | **Description** | **Reference** |
| --- | --- | --- |
| BL21 (DE3) | *E. coli* lab strain with no Lon and OmpT proteases | (8) |
| MG1655 | *E. coli* lab strain | (9) |
| NUH04 | K2 ST2039 KLA isolate | This study |
| NUH11 | K136 ST399 KLA isolate (*iroP* negative) | This study |
| NUH29 | K28 ST20 KLA isolate (*iroP* negative) | This study |
| SGH07 | K5 ST60 KLA isolate | This study |
| TTSH21 | K5 ST60 KLA isolate | This study |
| TTSH29 | K2 ST380 KLA isolate | This study |
| TTSH44 | K127 ST1307 KLA isolate (*iroP* negative) | This study |
| TTSH47 | K2 ST86 KLA isolate (*iroP* negative) | This study |
| TTSH64 | KL133 ST276-2LV KLA isolate (*iroP* negative) | This study |
| SGH10 | K1 CG23-I KLA isolate | (10, 11) |
| SGH10 Δ*Kp*VP | SGH10 large virulence plasmid *Kp*VP cured mutant | This study |
| SGH10 Δ*rmpA* | SGH10 with no hypermucoid capsule production | (7) |
| SGH10 Δ*wcaJ* | SGH10 capsule-null mutant | (7) |
| SGH10 Δ*mrkA* | SGH10 with no production of T3F major structural subunit | This study |
| SGH10 Δ*Kp*VP Δ*mrkA* | SGH10 *Kp*VP cured mutant with no T3F | This study |
| SGH10 Δ*Kp*VP Δ*wcaJ* | SGH10 capsule-null mutant with no *Kp*VP | This study |
| SGH10 Δ*iuc-rmp-iro* | SGH10 *Kp*VP partial deleted region comprising of aerobactin, *rmp* operon and salmochelin | This study |
| SGH10 Δ*iucCD* | SGH10 with impaired aerobactin synthesis | This study |
| SGH10 Δ*iro* | SGH10 with no salmochelin cluster | This study |
| SGH10 Δ*iroB* | SGH10 with no salmochelin glycosyltransferase on *Kp*VP | This study |
| SGH10 Δ*iroC* | SGH10 with no salmochelin periplasmic transporter | This study |
| SGH10 Δ*iroD* | SGH10 with no salmochelin iron esterase | This study |
| SGH10 Δ*iroN* | SGH10 with no salmochelin outer membrane receptor (including promoter region) | This study |
| SGH10 Δ*iroN* CDS | SGH10 with no salmochelin outer membrane receptor | This study |
| SGH10 Δ*mceC* Δ*iroB* | SGH10 with impaired salmochelin synthesis (lack of both glucosyltransferases in *Kp*VP and chromosomal GIE492) | This study |
| SGH10 ΔGIE492 | SGH10 with deleted chromosomal GIE492 (no microcin E492 production) | This study |
| SGH10 Δ*mceC* | SGH10 with no *iroB* homolog in chromosomal GIE492 | This study |
| SGH10 Δ*iroP* | SGH10 with deleted *iroP* | This study |
| SGH10 Δ*rmpA* Δ*iroP* | SGH10 hypermucoid negative mutant with deleted *iroP* | This study |
| SGH10 Δ*mrkA* Δ*iroP* | SGH10 hypermucoid negative mutant with deleted *mrkA* | This study |
| SGH10 Δ*rmpA* Δ*iroP* Δ*mrkA* | SGH10 hypermucoid negative mutant with deleted *iroP* and *mrkA* | This study |
| SGH10 Δ*fur* | SGH10 Ferric Uptake Regulator (Fur) deletion mutant | This study |
| SGH10 Δ*iroP* Δ*fur* | SGH10 with deleted *iroP* and *fur* | This study |
| SGH10::*iroP*-FLAG | SGH10 with endogenous FLAG tagged IroP in *Kp*VP | This study |
| SGH10 Δ*fur*::IroP-FLAG | SGH10 Δ*fur* mutant with endogenous FLAG tagged IroP in *Kp*VP | This study |
| SGH10 Δ*iroN* / pMLBAD::*iroN* CDS | SGH10 Δ*iroN* with *iroN* CDS complementation in pMLBAD | This study |
| SGH10 Δ*iroP* / pMLBAD | SGH10 Δ*iroP* with empty pMLBAD vector | This study |
| SGH10 Δ*iroP* / pMLBAD::*iroP* | SGH10 Δ*iroP* with *iroP* complementation in pMLBAD | This study |
| SGH10 Δ*Kp*VP / pMLBAD | SGH10 Δ*Kp*VP with empty pMLBAD vector | This study |
| SGH10 Δ*Kp*VP / pMLBAD::*iroP* | SGH10 Δ*Kp*VP with *iroP* complementation in pMLBAD | This study |
| SGH10 Δ*Kp*VP / pUCP28T | SGH10 Δ*Kp*VP with empty pUCP28T high copy plasmid | This study |
| SGH10 Δ*Kp*VP / pUCP28T::*iroP*-FLAG | SGH10 Δ*Kp*VP with IroP-FLAG in pUCP28T high copy plasmid | This study |
| BL21 (DE3) / pBAD33 / pUCP28T::*P_mrkA_*-sfGFP | BL21 (DE3) carrying empty pBAD33 and pUCP28T with sfGFP driven by *mrkA* promoter | This study |
| BL21 (DE3) / pBAD33 / pUCP28T::*P_mrkH_*-sfGFP | BL21 (DE3) carrying empty pBAD33 and pUCP28T with sfGFP driven by *mrkH* promoter | This study |
| BL21 (DE3) / pBAD33::*iroP* / pUCP28T::*P_mrkA_*-sfGFP | BL21 (DE3) carrying pBAD33 with arabinose inducible *iroP* and pUCP28T with sfGFP driven by *mrkH* promoter | This study |
| BL21 (DE3) / pBAD33:*iroP* / pUCP28T::*P_mrkH_*-sfGFP | BL21 (DE3) carrying pBAD33 with arabinose inducible *iroP* and pUCP28T with sfGFP driven by *mrkH* promoter | This study |
| SGH10 Δ*Kp*VP Δ*wcaJ* / pBAD33 / pUCP28T::*P_mrkA_*-sfGFP | SGH10 Δ*Kp*VP Δ*wcaJ* carrying empty pBAD33 and pUCP28T with sfGFP driven by *mrkH* promoter | This study |
| SGH10 Δ*Kp*VP Δ*wcaJ* / pBAD33 / pUCP28T::*P_mrkH_*-sfGFP | SGH10 Δ*Kp*VP Δ*wcaJ* carrying empty pBAD33 and pUCP28T with sfGFP driven by *mrkH* promoter | This study |
| SGH10 Δ*Kp*VP Δ*wcaJ* / pBAD33::*iroP* / pUCP28T::*P_mrkA_*-sfGFP | SGH10 Δ*Kp*VP Δ*wcaJ* carrying pBAD33 with arabinose inducible *iroP* and pUCP28T with sfGFP driven by  *mrkA* promoter | This study |
| SGH10 Δ*Kp*VP Δ*wcaJ* / pBAD33::*iroP* / pUCP28T::*P_mrkH_*-sfGFP | SGH10 Δ*Kp*VP Δ*wcaJ* carrying pBAD33 with arabinose inducible *iroP* and pUCP28T with sfGFP driven by *mrkH* promoter | This study |
| SGH10 Δ*wcaJ* / pPROBE | SGH10 Δ*wcaJ* carrying empty pPROBE-GFP tagless plasmid | This study |
| SGH10 Δ*wcaJ* / pPROBE::200 bp *iroP* | SGH10 Δ*wcaJ* carrying pPROBE with 200 bp upstream of *iroP* coding sequence and GFP | This study |
| SGH10 Δ*wcaJ* / pPROBE::*P_iroN_* | SGH10 Δ*wcaJ* carrying pPROBE with *iroN* promoter and GFP | This study |
| TTSH21 / pMLBAD::*iroP* | TTSH21 carrying SGH10 *iroP* in pMLBAD | This study |
| NUH29 / pMLBAD | NUH29 carrying empty pMLBAD vector | This study |
| NUH29 / pMLBAD::*iroP* | NUH29 carrying SGH10 *iroP* in pMLBAD | This study |

**Table S4. List of primers used for construction of deletion mutants and gene complementation.**

| **Primer Name** | **Sequence (5’-3’)** |
| --- | --- |
| *vagD* Up For | TATGACATGATTACGAATTCATCAGTGGCTCCGGTATGAC |
| *vagD* Up Rev | GGATCCCCGGGTACCGTGTCGAGCATCCAGGTTTT |
| *vagD* Down For | GGATCCTCTAGAGTCCGCTCTCTGCAGGGTAATTT |
| *vagD* Down Rev | CTTGCATGCCTGCAGCCCACCAATAGTGGACACAG |
| pSLC217 *relE* For | GGTACCCGGGGATCCTGAGCGATTGTGTAGGCTGG |
| pSLC217 *relE* Rev | GACTCTAGAGGATCCATCGTGAGGATGCGTCATCG |
| *iuc-rmpA-iro* Up For | ACATGATTACGAATTCTCTCCTTCAGCCCGAACAA |
| *iuc-rmpA-iro* Up Rev | CACAGAGTCAGCAGGACCACCTTTAGCA |
| *iuc-rmpA-iro* Dn For | TCCTGCTGACTCTGTGCTGTTCTGACCG |
| *iuc-rmpA-iro* Dn Rev | CTTGCATGCCTGCAGCGGTCACTTCGTCTAGCGTT |
| *iucCD* KO Up For | CTTGCATGCCTGCAGGATATTTATCGCCGCACCGC |
| *iucCD* KO Up Rev | CGTCGAAACTGCTCATACTCCAGCTCGG |
| *iucCD* KO Dn For | ATGAGCAGTTTCGACGAGAGACACCAGC |
| *iucCD* KO Dn Rev | ACATGATTACGAATTCTACGTGCAGATCTCCATGCC |
| *iro* KO Up For | TATGACATGATTACGAATTCCTCCGGGTGAGAAGAGACTG |
| *iro* KO Up Rev | TAAGACGAAGCCTCTTTTAACGCTCCTGTATACTA |
| *iro* KO Dn For | TTAAAAGAGGCTTCGTCTTAACATCGGTGTCAGT |
| *iro* KO Dn Rev | CTTGCATGCCTGCAGTGAAACTAGCGCAGAGGAAAATTAA |
| *iroB* KO Up For | TATGACATGATTACGAATTCTACCGGAGGTGGAGGTACTG |
| *iroB* KO Up Rev | CGTGCTGGCGTTTGTGTTTTGATTTTGCAATGGT |
| *iroB* KO Dn For | AAAACACAAACGCCAGCACGCATCCTAGA |
| *iroB* KO Dn Rev | CTTGCATGCCTGCAGGTTCCGAGATTGATCCGCCCAT |
| *iroC* KO Up For | TATGACATGATTACGAATTCCTGCCATAGTATACAGGAGCGTT |
| *iroC* KO Up Rev | CTAAAGCGGCGGTGTGGTGGCTACCCTTTC |
| *iroC* KO Dn For | CCACCACACCGCCGCTTTAGCTATTTATACTGTT |
| *iroC* KO Dn Rev | CTTGCATGCCTGCAGCTGAAAGTATTCTCGGGTCAAGT |
| *iroD* KO Up For | TATGACATGATTACGAATTCTTTATCGCCGACTCAGCTCC |
| *iroD* KO Up Rev | GCCATATCTAGTGCACTGTAACTATCCCGC |
| *iroD* KO Dn For | TACAGTGCACTAGATATGGCTCATGGCGTAAGG |
| *iroD* KO Dn Rev | CTTGCATGCCTGCAGGGCTTCGAAATCGAGAATTTGC |
| *iroN* (with promoter) KO Up For | TATGACATGATTACGAATTCGAATGCTGAACATGCAACAAC |
| *iroN* (with promoter) KO Up Rev | GAAAAAGCTTTAGCCTTACGCCATGAGCC |
| *iroN* (with promoter) KO Dn For | CGTAAGGCTAAAGCTTTTTCAATACCCTGAGGATG |
| *iroN* (with promoter) KO Dn Rev | CTTGCATGCCTGCAGGCTGTAACTTGGGGAACCACG |
| *iroN* CDS KO Up For | TATGACATGATTACGAATTCCGCATCGTTCCCGTAGCTATAA |
| *iroN* CDS KO Up Rev | ATAATTACGAACCCATTCCCTAATGAATGCTTAAC |
| *iroN* CDS KO Dn For | GGGAATGGGTTCGTAATTATTAGGACTAAGCAAGAT |
| *iroN* CDS KO Dn Rev | CTTGCATGCCTGCAGGAGTTATTGCCCTTTCTTCTGC |
| *iroP* KO Up For | TATGACATGATTACGAATTCATAATATTGAGCCGGTTCCC |
| *iroP* KO Up Rev | ATGAGGTTTAAATCGATCCTCAAGATTTTATATAACA |
| *iroP* KO Dn For | AGGATCGATTTAAACCTCATCGCATCCGCAAG |
| *iroP* KO Dn Rev | CTTGCATGCCTGCAGGCTGTAACTTGGGGAACCAC |
| *mrkA* KO up For | TATGACATGATTACGAATTCGGTGTACGCTTCGGTAATGT |
| *mrkA* KO up Rev | CCCCATCGCGTGCCATTTCCTTGTCAGAGTG |
| *mrkA* KO Dn For | GGAAATGGCACGCGATGGGGCAGTTAATAA |
| *mrkA* KO Dn Rev | CTTGCATGCCTGCAGTGTTAACGGTCAGGGTTTCG |
| *mceC* KO Up For | ACATGATTACGAATTCGGGATGGGGACATTTACAGGACC |
| *mceC* KO Up Rev | TGTTATACAAAAGAAAATCCTTCTCCGTTTTAGGAACTT |
| *mceC* KO Dn For | GGATTTTCTTTTGTATAACACTGCTGAAATTATTCACGAAAGC |
| *mceC* KO Dn Rev | CTTGCATGCCTGCAGGAATCAGTCCTTTCTCTTGATCTGGCTC |
| *fur* KO Up For | TATGACATGATTACGAATTCAAACCCGCATTCATGTAGCA |
| *fur* KO Up Rev | AAACGGGGAAGCGGAATCTGTCCTGTTGCT |
| *fur* KO Dn For | CAGATTCCGCTTCCCCGTTTCGCTGAGA |
| *fur* KO Dn Rev | CTTGCATGCCTGCAGCACCTCTGGGAGAACGACAA |
| *iroN* CDS For | TAGCAGGAGGAATTCCGATCAACCACTTTTAAGTTAAGCA |
| *iroN* CDS Rev | CTTGCATGCCTGCAGCGATCAGAATGAAACTACCGCC |
| *iroP* pMLBAD For | TAGCAGGAGGAATTCTCGTAATTATTAGGACTAAGCAAGAT |
| *iroP* pMLBAD Rev | CTTGCATGCCTGCAGTCTTTTAAATTTATTTGTTATCTGC |
| *iroP* pBAD33 For | GGAGATATACATATGTCGTAATTATTAGGACTAAGCAAGAT |
| *iroP* pBAD33 Rev | GATGGATATCTGCAGTCTTTTAAATTTATTTGTTATCTGC |
| *P_mrkA_* For | CTTGCATGCCTGCAGGGTGTTTGTCCTTTTAACTTTT |
| *P_mrkA_* Rev | GATCCCCGGGTACCGTGCCATTTCCTTGTCAGAGTGA |
| *P_mrkH_* For | CTTGCATGCCTGCAGCCTGCCAGACAAAATGGAGG |
| *P_mrkH_* Rev | GATCCCCGGGTACCGATGCATCCCTTGTAAATAGTTGTCG |
| sfGFP with *P_mrkA_* For | GACAAGGAAATGGCACGGTACCCGGGGATCCTCTA |
| sfGFP with *P_mrkH_* For | TTACAAGGGATGCATCGGTACCCGGGGATCCTCTA |
| sfGFP Rev | CATGATTACGAATTCAGGCTGAAAATCTTCTCTCATCCG |
| 1X FLAG insertion to *iroP* For | TTACTTGTCGTCATCGTCTTTGTAGTCACCTCTAGCACGGTTCAAAT |
| 1X FLAG insertion to *iroP* Rev | GACTACAAAGACGATGACGACAAGTAATAAACCTCATCGCATCCGCAAG |
| *iroP* 200 bp upstream pPROBE For | GGATCCCCGGGTACCCGATATTAATAAAAATCTTCGTCTT |
| *iroP* pPROBE Rev | TTAGTTAGGGAATTCTTAACCTCTAGCACGGTTCA |
| *P_iroN_* promoter pPROBE For | GGATCCCCGGGTACCCTTGACCCGAGAATACTTTCAGACA |
| *P_iroN_* promoter pPROBE Rev | TTAGTTAGGGAATTCACCCATTCCCTAATGAATGCTTAAC |

**Table S5. List of primers used for real-time qPCR in this study.**

| **qPCR Primer Name** | **Sequence (5’-3’)** |
| --- | --- |
| *recA* RT1 | AAATCGGTCAGGGTAAAGCG |
| *recA* RT2 | ATTGTTGAGCAGCAGTTCGC |
| *rpoB* RT1 | TTTCACTGCTGCGGAAATCG |
| *rpoB* RT2 | AATGGCGGAAAACCAGTTCC |
| *mrkA* RT1 | GCGGCCAGGTTAATTTCTTC |
| *mrkA* RT2 | ACAGGTAAACGTTCGCATCG |
| *mrkH* RT1 | CTCATGGCGTAAACGAAAGC |
| *mrkH* RT2 | ACATGACAATAGCGGTGTCG |
| *parA1* RT1 | AGGGCGGAATCGTTAAATCG |
| *parA1* RT2 | ATTTCAAGCACGCGGAAACC |
| *copC* RT1 | TCGTTATGGGCGTTGCTTTC |
| *copC* RT2 | CACCTGAGAATTTCACGGTCAG |
| *tRNA* RT1 | TTGACGAAGAAGCGTTGGAC |
| *tRNA* RT2 | TCCCATGCGTTTTTCAGGAC |
| *dppC* RT1 | ATGTTCGGAATGCTGTGTGC |
| *dppC* RT2 | AGCAACAGCGACAATTTCCC |
| *rmpA* RT1 | TTCAGGGAAATGGGGAGGGTA |
| *rmpA* RT2 | AAACGTCAAGCCACATCCATTG |
| *terC* RT1 | TCTTTGCCGTTGTTGTTGCC |
| *terC* RT2 | ATGCATGTCCGGACAGTTTC |
| *coA* RT1 | TCGCATCTGCTGTTTACGTC |
| *coA* RT2 | AGCCGAAAGAGATGCACAAG |
| *iroN* RT1 | GGCGATACGCAAAACAGTTC |
| *iroN* RT2 | TTGTCCCCAGTCCCAAATAC |
| *iroP* RT1 | GCTTTTTCAATACCCTGAGGATG |
| *iroP* RT2 | AGCGGCCTAAACCCATTATC |

**References**

1. Trapnell C, Williams BA, Pertea G, Mortazavi A, Kwan G, van Baren MJ, Salzberg SL, Wold BJ, Pachter L. 2010. Transcript assembly and quantification by RNA-Seq reveals unannotated transcripts and isoform switching during cell differentiation. Nature biotechnology 28:511-515.

2. Anders S, Huber W. 2010. Differential expression analysis for sequence count data. Genome Biology 11:R106.

3. Arora NK, Verma M. 2017. Modified microplate method for rapid and efficient estimation of siderophore produced by bacteria. 3 Biotech 7:381.

4. Aguilera P, Marcoleta A, Lobos-Ruiz P, Arranz R, Valpuesta JM, Monasterio O, Lagos R. 2016. Identification of Key Amino Acid Residues Modulating Intracellular and *in vitro* Microcin E492 Amyloid Formation. Frontiers in Microbiology 7:35.

5. Marcoleta AE, Gutiérrez-Cortez S, Hurtado F, Argandoña Y, Corsini G, Monasterio O, Lagos R. 2018. The Ferric uptake regulator (Fur) and iron availability control the production and maturation of the antibacterial peptide microcin E492. PLOS ONE 13:e0200835.

6. Nolan EM, Fischbach MA, Koglin A, Walsh CT. 2007. Biosynthetic Tailoring of Microcin E492m: Post-Translational Modification Affords an Antibacterial Siderophore-Peptide Conjugate. Journal of the American Chemical Society 129:14336-14347.

7. Tan YH, Chen Y, Chu WHW, Sham LT, Gan YH. 2020. Cell envelope defects of different capsule‐null mutants in K1 hypervirulent *Klebsiella pneumoniae* can affect bacterial pathogenesis. Molecular Microbiology 113:889-905.

8. Studier FW, Moffatt BA. 1986. Use of bacteriophage T7 RNA polymerase to direct selective high-level expression of cloned genes. J Mol Biol 189:113-30.

9. Guyer MS, Reed RR, Steitz JA, Low KB. 1981. Identification of a sex-factor-affinity site in *E. coli* as gamma delta. Cold Spring Harb Symp Quant Biol 45:135-140.

10. Lam MMC, Wyres KL, Duchêne S, Wick RR, Judd LM, Gan Y-H, Hoh C-H, Archuleta S, Molton JS, Kalimuddin S, Koh TH, Passet V, Brisse S, Holt KE. 2018. Population genomics of hypervirulent *Klebsiella pneumoniae* clonal-group 23 reveals early emergence and rapid global dissemination. Nature Communications 9:2703.

11. Lee IR, Molton JS, Wyres KL, Gorrie C, Wong J, Hoh CH, Teo J, Kalimuddin S, Lye DC, Archuleta S, Holt KE, Gan Y-H. 2016. Differential host susceptibility and bacterial virulence factors driving *Klebsiella* liver abscess in an ethnically diverse population. Scientific Reports 6:29316.
